# Dysregulation of INF2-mediated mitochondrial fission in SPOP-mutated prostate cancer

**DOI:** 10.1371/journal.pgen.1006748

**Published:** 2017-04-27

**Authors:** Xiaofeng Jin, Jie Wang, Kun Gao, Pingzhao Zhang, Longfang Yao, Yan Tang, Lisha Tang, Jian Ma, Jiantao Xiao, Enceng Zhang, Jie Zhu, Bin Zhang, Shi-min Zhao, Yao Li, Shancheng Ren, Haojie Huang, Long Yu, Chenji Wang

**Affiliations:** 1 State Key Lab of Genetic Engineering, School of Life Sciences, Fudan University, Shanghai, P.R. China; 2 Shanghai Institute of Planned Parenthood Research Hospital, WHO Collaborating Center for Research in Human Reproduction, Shanghai, P.R. China; 3 Fudan University Cancer Institute, Shanghai Cancer Center, Shanghai, P.R. China; 4 Department of Urology, Shanghai First People's Hospital, School of Medicine, Shanghai Jiaotong University, Shanghai, China; 5 Department of hepato-biliary-pancreatic Surgery, Ningbo Medical Center of LiHuiLi Hospital, Ningbo, P.R. China; 6 Department of Urology, Shanghai Changhai Hospital, Second Military Medical University, Shanghai, P.R. China; 7 Department of Biochemistry and Molecular Biology, Mayo Clinic College of Medicine, Rochester, Minnesota, United States of America; St Jude Children's Research Hospital, UNITED STATES

## Abstract

Next-generation sequencing of the exome and genome of prostate cancers has identified numerous genetic alternations. SPOP (Speckle-type POZ Protein) was one of the most frequently mutated genes in primary prostate cancer, suggesting SPOP is a potential driver of prostate cancer development and progression. However, how SPOP mutations contribute to prostate cancer pathogenesis remains poorly understood. SPOP acts as an adaptor protein of the CUL3-RBX1 E3 ubiquitin ligase complex that generally recruits substrates for ubiquitination and subsequent degradation. ER-localized isoform of the formin protein inverted formin 2 (INF2) mediates actin polymerization at ER-mitochondria intersections and facilitates DRP1 recruitment to mitochondria, which is a critical step in mitochondrial fission. Here, we revealed that SPOP recognizes a Ser/Thr (S/T)-rich motif in the C-terminal region of INF2 and triggers atypical polyubiquitination of INF2. These ubiquitination modifications do not lead to INF2 instability, but rather reduces INF2 localization in ER and mitochondrially associated DRP1 puncta formation, therefore abrogates its ability to facilitate mitochondrial fission. INF2 mutant escaping from SPOP-mediated ubiquitination is more potent in prompting mitochondrial fission. Moreover, prostate cancer-associated SPOP mutants increase INF2 localization in ER and promote mitochondrial fission, probably through a dominant-negative effect to inhibit endogenous SPOP. Moreover, INF2 is important for SPOP inactivation-induced prostate cancer cell migration and invasion. These findings reveal novel molecular events underlying the regulation of INF2 function and localization, and provided insights in understanding the relationship between SPOP mutations and dysregulation of mitochondrial dynamics in prostate cancer.

## Author summary

Prostate cancer is the leading cause of global cancer-related death. The development of improved diagnoses and novel therapies has been confounded by significant patient heterogeneity. During recent years, significant progress has been made in identifying the molecular alterations in prostate cancer using next-generation sequencing. SPOP gene was frequently altered by somatic point mutations in a distinct molecular subclass of prostate cancer, although the precise role that SPOP mutation plays in the development of prostate cancer is unclear. Mitochondria are highly motile organelles that undergo constant fission and fusion. Unbalanced mitochondrial fission and fusion events are associated with mitochondrial dysfunction and frequently linked to human cancer. Here, we are the first to report that SPOP mutations are associated with dysregulation of mitochondrial dynamics in prostate cancer and this finding may have potential clinical implications in prostate cancer treatment.

## Introduction

Large-scale exome/genome sequencing studies have recently revealed that recurrent mutations in the *SPOP* gene occur in up to 15% of prostate cancers [1–4]. Interestingly, the SPOP mutant subset of prostate cancers had some notable molecular features, including mutual exclusivity with *ERG* gene rearrangement, elevated levels of DNA methylation, homogeneous gene expression patterns, frequent deletion of *CHD1* and overexpression of *SPINK1* mRNA, supporting the concept that SPOP mutation tumors represent a distinct molecular subclass of prostate cancer [4] SPOP is one of the adaptor proteins of the CUL3-RBX1 E3 ubiquitin ligase complexes. It selectively recruits substrates via its N-terminal MATH domain, whereas its BTB and BACK domains mediate oligomerization and interaction with CUL3 [5]. SPOP has been linked to the ubiquitination and degradation of several substrates, including the steroid receptor coactivator 3 (SRC-3), androgen receptor (AR), DEK, ERG, SENP7 and several others [6–11]. All prostate cancer-associated SPOP mutations identified so far affect evolutionarily conserved residues in the MATH domain, suggesting that these mutations may alter the interaction of SPOP with its substrates [1–4]. Inactivation of SPOP by knockdown or overexpression of prostate cancer-associated SPOP mutants leads to increased prostate cancer cell proliferation, migration and invasion, implying SPOP is a tumor suppressor [2,8–10]. However, limited numbers of SPOP substrates have been identified and functionally explored.

Mitochondria are highly motile organelles that undergo constant fission and fusion, and are actively transported to specific subcellular locations [12]. Unbalanced mitochondrial fission and fusion events are associated with mitochondrial dysfunction and frequently linked to the pathogenesis of many human diseases, including cancer [12,13]. The majority of studies that have explored mitochondrial morphology in tumor cells support a pro-tumorigenic role for mitochondrial fission and tumor suppressor role for mitochondrial fusion [14]. Mitochondrial fragmentation has been observed in various types of tumor cells [15–17]. Inhibition of mitochondrial fission decreases cell proliferation, migration and invasion in various cancer models including lung, colon, breast, thyroid cancer and glioblastoma[16–20]. While cancer is a disease characterized by multiple genetic aberrations, little is known about whether cancer-associated mutations can directly affect mitochondrial dynamics, and how this impacts upon tumor phenotypes.

Inverted formin 2 (INF2) is a unique vertebrate formin protein that accelerates both actin polymerization and depolymerization [21]. In mammalian cells, INF2 can be expressed as two C-terminal splice variants: the prenylated (CAAX) isoform, which is tightly bound to endoplasmic reticulum (ER) [22], and the nonCAAX isoform, which is cytoplasmic [23]. Recent studies have persuasively showed in mammalian cells that actin polymerization mediated by ER-localized INF2 CAAX isoform is required for mitochondrial fission [24]. By contrast, the cellular function of the nonCAAX isoform of INF2 has been less characterized. Suppression of INF2-nonCAAX isoform in cells causes Golgi dispersal, suggesting INF2 might be involved in maintenance of Golgi architecture [23]. Mutations in INF2 are linked to two human genetic diseases: focal and segmental glomerulosclerosis (FSGS), a degenerative kidney disease [25], and Charcot-Marie-Tooth disease (CMTD), a neurological disorder [26]. However, little is known about how INF2 protein is physiologically regulated.

In this study, we demonstrate that SPOP suppresses mitochondrial fission by promoting atypical ubiquitination and relocalization of ER-localized INF2. Moreover, this effect is abrogated by the prostate cancer-associated SPOP mutations. Thus, our results provide a functional link between SPOP mutations and dysregulation of mitochondrial dynamics in prostate cancer.

## Results

### Identification of INF2 as a novel SPOP Interactor

**Fig 1:**
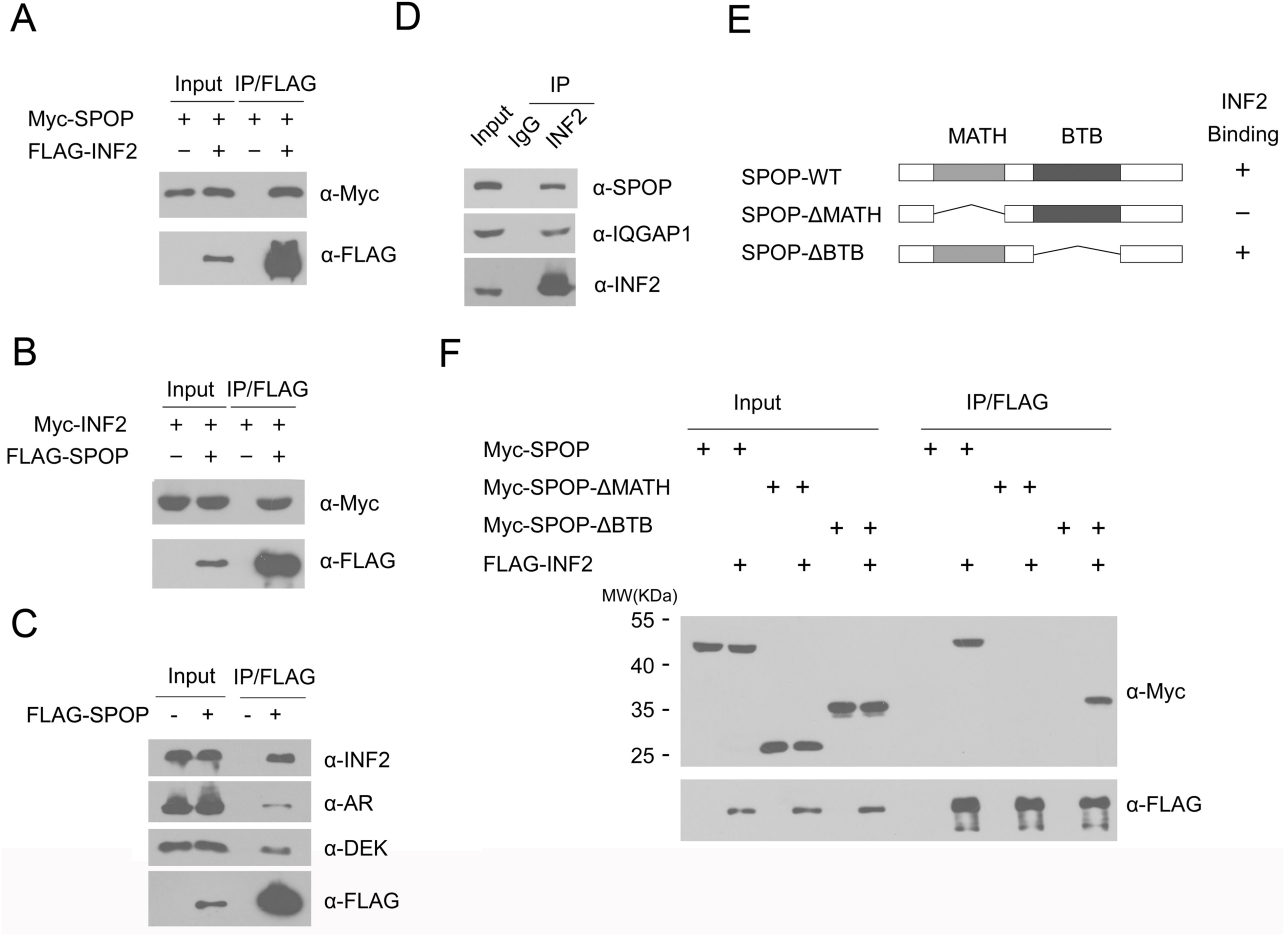
SPOP interacts with INF2 protein in cells. (A) Western blot of whole cell lysates (WCL) and co-IP samples of anti-FLAG antibody obtained from 293T cells transfected with indicated plasmids. (B) Western blot of WCL and co-IP samples of anti-Myc antibody obtained from 293T cells transfected with indicated plasmids. (C) Western blot of WCL and co-IP samples of anti-FLAG antibody obtained from LNCaP cells infected with lentivirus expressing FLAG-SPOP or control. The cells were treated with 20 μM MG132 for 8 h before harvesting. (D) Western blot of co-IP samples of IgG or anti-INF2 antibodies obtained from cell lysates of LNCaP cells. (E) Schematic representation of SPOP deletion mutants. Binding capacity of SPOP to INF2 is indicated with the symbol. (F) Western blot of WCL and co-IP samples of anti-FLAG antibody obtained from 293T cells transfected with indicated plasmids.

To identify molecular mediators of the tumor suppressive function of SPOP, we performed a yeast two-hybrid screen in a human fetal brain cDNA library using the full length SPOP as bait. Among the positive clones identified, 4 clones were INF2 fragments. Considering INF2 is an important regulator of actin polymerization and mitochondrial fission, we explored whether INF2 is an authentic SPOP substrate and its function is dysregulated in SPOP-mutated prostate cancer. We first examined whether SPOP interacts with INF2 in cells. To do this, FLAG-INF2, and Myc-SPOP were co-expressed in 293T cells. Cell lysates were subsequently prepared for co-immunoprecipitation (co-IP) with anti-FLAG antibody. As shown in Fig 1A, Myc-SPOP was immunoprecipitated by FLAG-INF2, suggesting an interaction between SPOP and INF2 proteins. Similar results were also obtained in the reciprocal co-IP experiment in which FLAG-SPOP was able to immunoprecipitate Myc-INF2(Fig 1B). FLAG-SPOP was able to immunoprecipitate endogenous INF2, and two known SPOP substrates (AR and DEK) in LNCaP cells (Fig 1C). Next, we decided to extend our analysis by investigating whether endogenous SPOP and INF2 can interact with each other in prostate cancer cells. Immunoprecipitation using anti-INF2 antibody was performed using cell lysates prepared from LNCaP cells. As shown in Fig 1D, INF2 was able to immunoprecipitate SPOP and a known interactor IQGAP1, suggesting that SPOP can interact with INF2 protein at endogenous level.

SPOP contains two structural domains: a substrate-binding MATH domain at the N-terminus and a CUL3-binding BTB domain at the C-terminus. To determine which domain may mediate its interaction with INF2, we generated two deletion mutants of SPOP (SPOP-ΔBTB and ΔMATH), corresponding to the deletion of these two domains respectively (Fig 1E). Co-IP assay was performed to examine the binding of INF2 with the full length SPOP (SPOP-WT) and the two deletion mutants. As shown in Fig 1F, SPOP-WT and SPOP-ΔBTB, but not SPOP-ΔMATH interacted with INF2. Therefore, our findings demonstrate that SPOP binds INF2 via the MATH domain.

### SPOP promotes INF2 ubiquitination, but not degradation

**Fig 2:**
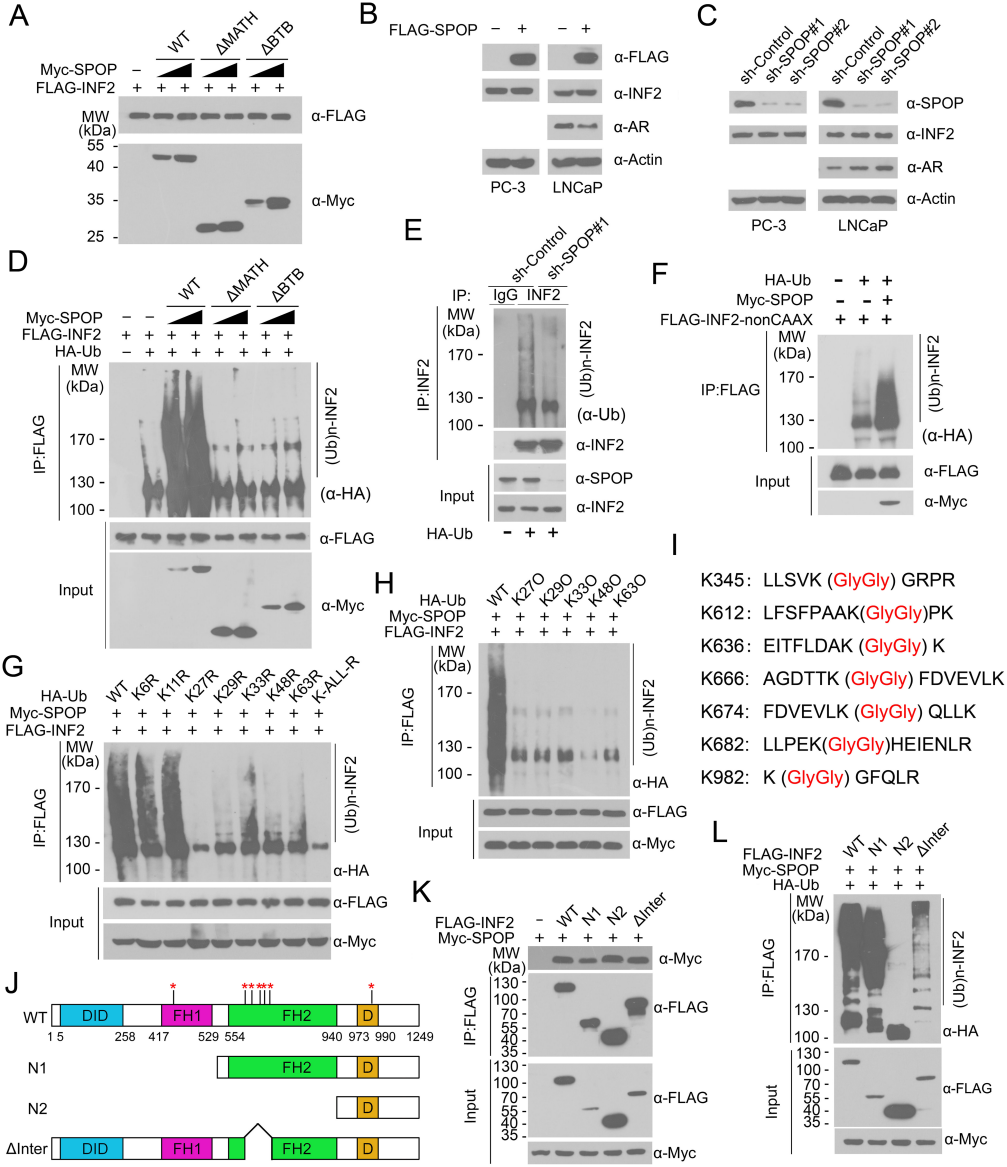
SPOP promotes INF2 protein ubiquitination but not degradation. (A) Western blot of WCL from 293T cells transfected with indicated plasmids. (B) Western blot of WCL from LNCaP or DU145 cells infected with lentivirus expressing FLAG-SPOP or control. (C) Western blot of WCL of LNCaP or DU145 cells infected with lentivirus expressing SPOP-specific shRNA or scramble control. (D) Western blot of the products of *in vivo* ubiquitination assay performed using cell lysates form 293T cells transfected with indicated plasmids. (E) Western blot of the products of *in vivo* ubiquitination assay performed using cell lysates from LNCaP cells transfected with HA-Ub and indicated shRNAs. (F) Western blot of the products of *in vivo* ubiquitination assay performed using cell lysates from 293T cells transfected with indicated plasmids. (G) Western blot of the products of *in vivo* ubiquitination assay performed using cell lysates from 293T cells transfected with FLAG-INF2, Myc-SPOP and HA-Ub WT or mutants carrying a single K/R substitution, as indicated. (H) *In vivo* ubiquitination assay was performed as in (G) by using the K-only set of HA-Ub. (i) Identification of ubiquitin attachment sites on INF2 (see Materials and methods for details). (J) Schematic representation of INF2 domain architecture and its deletion mutants. Red asterisk indicates ubiquitin attachment sites. (K) Western blot of WCL and co-IP samples of anti-FLAG antibody obtained from 293T cells transfected with indicated plasmids. (I) Western blot of the products of in *vivo* ubiquitination assay performed using cell lysates from 293T cells transfected with indicated plasmids.

We then explored whether SPOP can promote the ubiquitination and degradation of INF2. Unexpectedly, overexpression of wild-type SPOP or its mutants (SPOP-ΔBTB, ΔMATH) did not alter the protein level of ectopically co-expressed INF2 (Fig 2A). Moreover, we found that ectopic expression of SPOP in LNCaP or DU145 prostate cancer cells did not alter the protein level of endogenous INF2 (Fig 2B). In contrast, SPOP overexpression in LNCaP cells (AR positive) decreased the expression of endogenous AR, a known SPOP substrate (Fig 2B) [7,27]. Consistent with these findings, depletion of endogenous SPOP by two independent shRNAs did not alter INF2 protein level in both LNCaP and DU145 (AR-negative) cells, but elevated AR protein level in LNCaP cells (Fig 2C). Thus, these results demonstrate that SPOP does not affect INF2 protein level. To determine whether SPOP regulates INF2 polyubiquitination, HA-Ub and FLAG-INF2 were co-expressed in 293T cells with increasing doses of SPOP-WT or its mutants (SPOP-ΔBTB, ΔMATH). As shown in Fig 2D, INF2 protein was robustly polyubiquitinated by co-expression of SPOP-WT, but not SPOP-ΔBTB or ΔMATH, in a dose-dependent manner. Accordingly, depletion of SPOP in LNCaP cells decreased the ubiquitination of endogenous INF2 (Fig 2E). Since the INF2 construct used in above analysis is the CAAX isoform, we examined whether SPOP can ubiquitinate the nonCAAX isoform. As shown Fig 2F, INF2 nonCAAX isoform was also robustly polyubiquitinated by SPOP. Taken together, our data suggest that SPOP can promote INF2 ubiquitination, but not degradation.

We then examined the linkage specificity of SPOP-mediated INF2 ubiquitination. *In vivo* ubiquitination assay was performed using a panel of ubiquitin mutants containing a single K/R mutation on each of the seven lysines in the ubiquitin sequence, potentially involved in the formation of polyUb chains. We also included a lysine-free ubiquitin mutant (K-ALL-R), in which all of the lysines were replaced with arginines. As shown in Fig 2G, expression of the K-ALL-R mutant abolished SPOP-mediated INF2 ubiquitination, excluding the possibility that SPOP promotes multiple mono-ubiquitination of INF2. Expression of K6R or K11R mutant marginally altered the amount of ubiquitinated INF2 (Fig 2G), suggesting that K6 and K11 are largely dispensable for SPOP-mediated INF2 ubiquitination. By contrast, a significant reduction of INF2 ubiquitination is instead observed when other ubiquitin mutants, including K27R, K29R, K33R, K48R and K63R, were used (Fig 2G). We next used a reciprocal series of mutants, where all the seven lysines in ubiquitin were converted to arginine residues, except one (one-K-Only mutants). As shown in Fig 2H, expression of K27O, K29O, K33O, K48O or K63O mutants completely abolished SPOP-mediated INF2 ubiquitination. Therefore, these data indicate that SPOP catalyzes synthesis of mixed-linkage polyUb chains on INF2, and K27, K29, K33, K48 and K63 residues in Ub are all essentially involved.

Having established that SPOP promotes atypical ubiquitination of INF2, we set out to identify the ubiquitin attachment sites on INF2. We co-expressed the FLAG-INF2, Myc-SPOP and HA-Ub constructs in 293T cells, and the immunoprecipitated ubiquitin-INF2 conjugates were analyzed by liquid chromatography tandem mass spectrometry (LC-MS/MS). It revealed that INF2 was ubiquitinated at least at 7 lysine residues (Fig 2I). Interestingly, 5 of 7 ubiquitin attachment sites are localized in a short stretch of sequence (amino acids 612–682) within the FH2 domain of INF2 (Fig 2J). To evaluate whether this region is important for INF2 ubiquitination, we constructed a series of INF2 deletion mutants and performed *in vivo* ubiquitination assay. While these deletion mutants were capable of binding to SPOP in a manner similar to the full length INF2 (Fig 2K), the N2 and ΔInter mutants, which lack the 612–682 aa region, were much less ubiquitinated by SPOP (Fig 2l). These data suggest that the lysine residues located in the 612–682 aa of INF2 serve as the predominant ubiquitin attachment sites.

### A SPOP-binding consensus motif (SBC) in INF2 is required for SPOP-mediated INF2 ubiquitination

**Fig 3:**
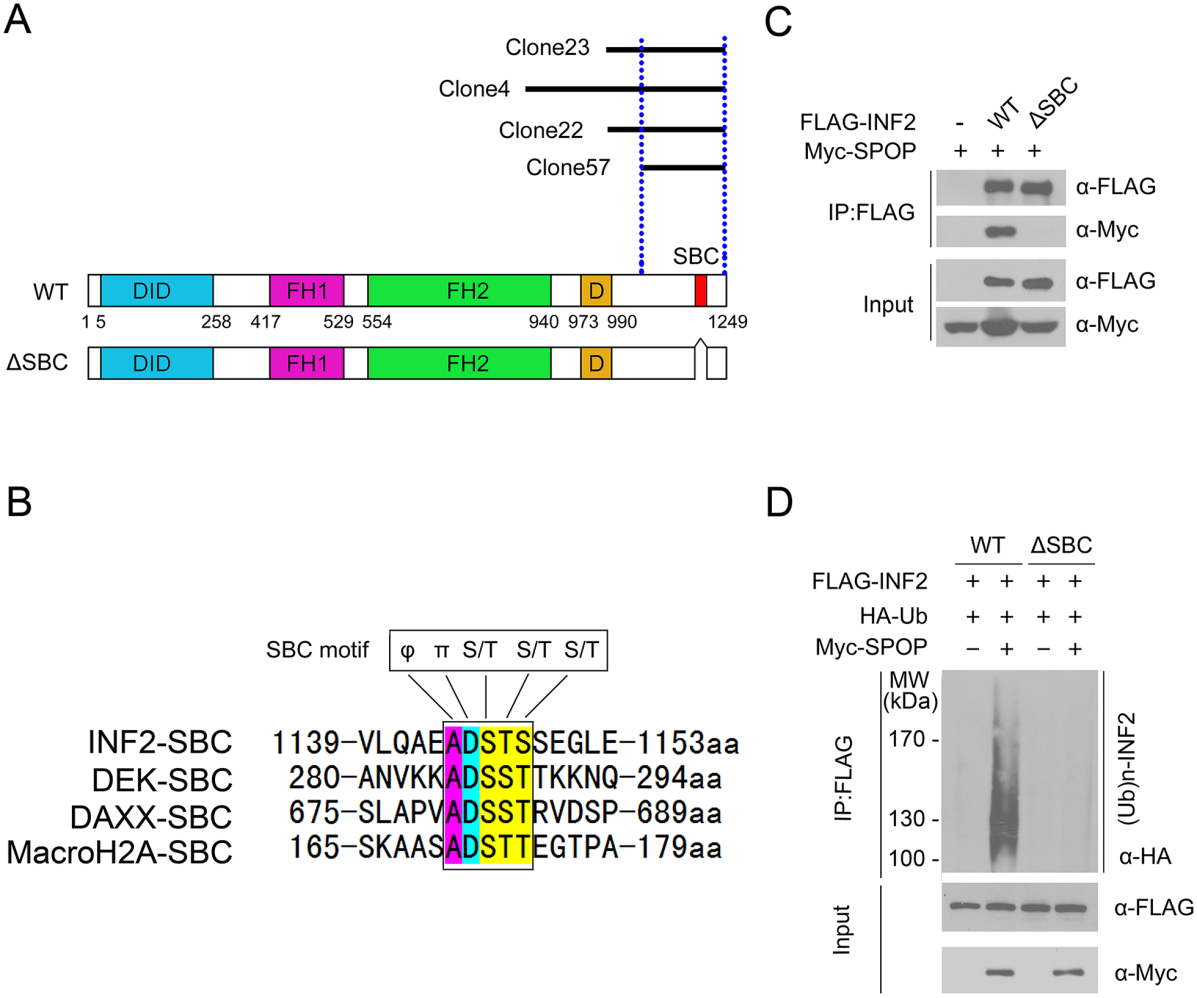
The SBC motif in INF2 is recognized by SPOP. (A) Diagram showing a putative SBC motif in INF2. It also shows the INF2 fragments identified in Y2H screening. (B) Alignment of the SBC motif in INF2 with other known SPOP substrates. (C) Western blot of WCL and co-IP samples of anti-FLAG antibody obtained from 293T cells transfected with indicated plasmids. (D) Western blot of the products of *in vivo* ubiquitination assay performed using cell lysates from 293T cells transfected with indicated plasmids.

Previous studies reported that one or several SBC motifs (Φ-π-S-S/T-S/T; Φ: nonpolar residues, π: polar residues) are present in known SPOP substrates [6–11,28]. We examined the protein sequence of INF2 that is required for SPOP-binding. To this end, we first deduced the minimal interacting region from the four INF2 fragments obtained in yeast two-hybrid screen. We found INF2 (1024~1249 aa) corresponds to the smallest region necessary for SPOP interaction (Fig 3A). Next we performed a protein motif search in the C-terminal region of INF2 and discovered a perfectly matched SBC motif (Fig 3A). Moreover, this motif is very similar to the SBC motifs present in MacroH2A, DAXX and DEK (Fig 3B). To examine whether this potential motif is actually required for SPOP-INF2 interaction, we generated an INF2 mutant in which the motif sequence was deleted. 293T cells were co-transfected with SPOP and wild-type INF2 or ΔSBC mutant. Co-IP assay demonstrated that SPOP only bound to the wild-type INF2, but not the ΔSBC mutant although they were expressed at comparable levels (Fig 3C), suggesting that the SBC motif of INF2 was required for SPOP binding. *In vivo* ubiquitination assay demonstrated that deletion of the SBC motif totally abolished SPOP-mediated INF2 ubiquitination (Fig 3D). Collectively, we have identified a conserved SBC motif present in INF2 that is indispensable for SPOP-INF2 interaction.

### Prostate cancer-associated mutants of SPOP are defective in promoting INF2 ubiquitination

**Fig 4:**
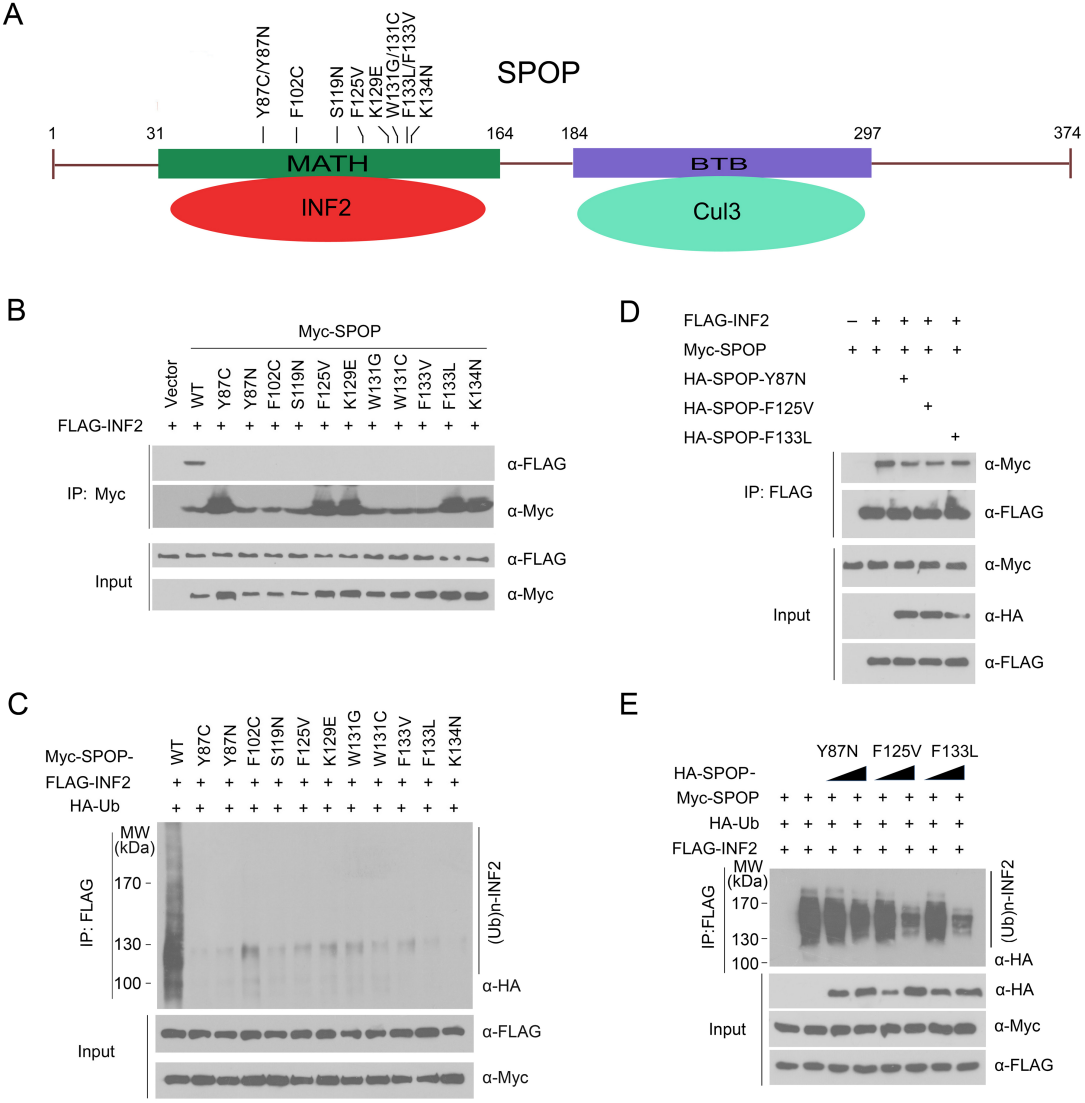
Prostate cancer-associated SPOP mutants cannot bind to and promote INF2 ubiquitination. (A) Distribution of the point mutations on the SPOP gene found in prostate cancer samples. (B) Western blot of WCL and co-IP samples of anti-FLAG antibody obtained from 293T cells transfected with indicated plasmids. (C) Western blot of the products of *in vivo* ubiquitination assay of 293T cells transfected with indicated plasmids. (D) Western blot of WCL and co-IP samples of anti-FLAG antibody obtained from 293T cells transfected with indicated plasmids. (E) Western blot of the products of *in vivo* ubiquitination assay of 293T cells transfected with indicated plasmids.

All the SPOP mutations detected thus far in prostate cancers exclusively occur in the MATH domain, which is responsible for substrate binding (Fig 4A). We postulated that prostate cancer-associated mutants of SPOP may be defective in mediating INF2 polyubiquitination. To test this, we generated a series of Myc-tagged prostate cancer-associated mutants of SPOP, including Y87C, Y87N, F102C, S119N, F125V, K129E, W131G, W131C, F133L, F133V and K134N, and examined their interactions with INF2 by co-IP assays. As shown in Fig 4B, mutations of the residues at the MATH domain abrogated the ability of SPOP to interact with INF2. Moreover, *in vivo* ubiquitination assay indicated that prostate cancer-associated SPOP mutants largely lost the capacity to promote INF2 polyubiquitination (Fig 4C).

Previous study showed that only one copy of SPOP allele is mutated in prostate cancer and SPOP mutants exert their tumor-promoting function in a dominant-negative manner to inhibit the wild-type SPOP [2]. We hypothesized that prostate cancer-associated mutations of SPOP might disrupt the interaction between wild-type SPOP and INF2. Indeed, we found that co-expression of SPOP mutants (Y87N, F125V or F133L) reduced the interaction between wild-type SPOP and INF2 (Fig 4D). Moreover, co-expression of SPOP mutants suppressed wild-type SPOP-induced INF2 ubiquitination (Fig 4E). Taken together, our findings suggest that INF2 ubiquitination may be dysregulated by oncogenic prostate cancer-associated SPOP mutants.

### SPOP induces INF2 disassociation from ER

**Fig 5:**
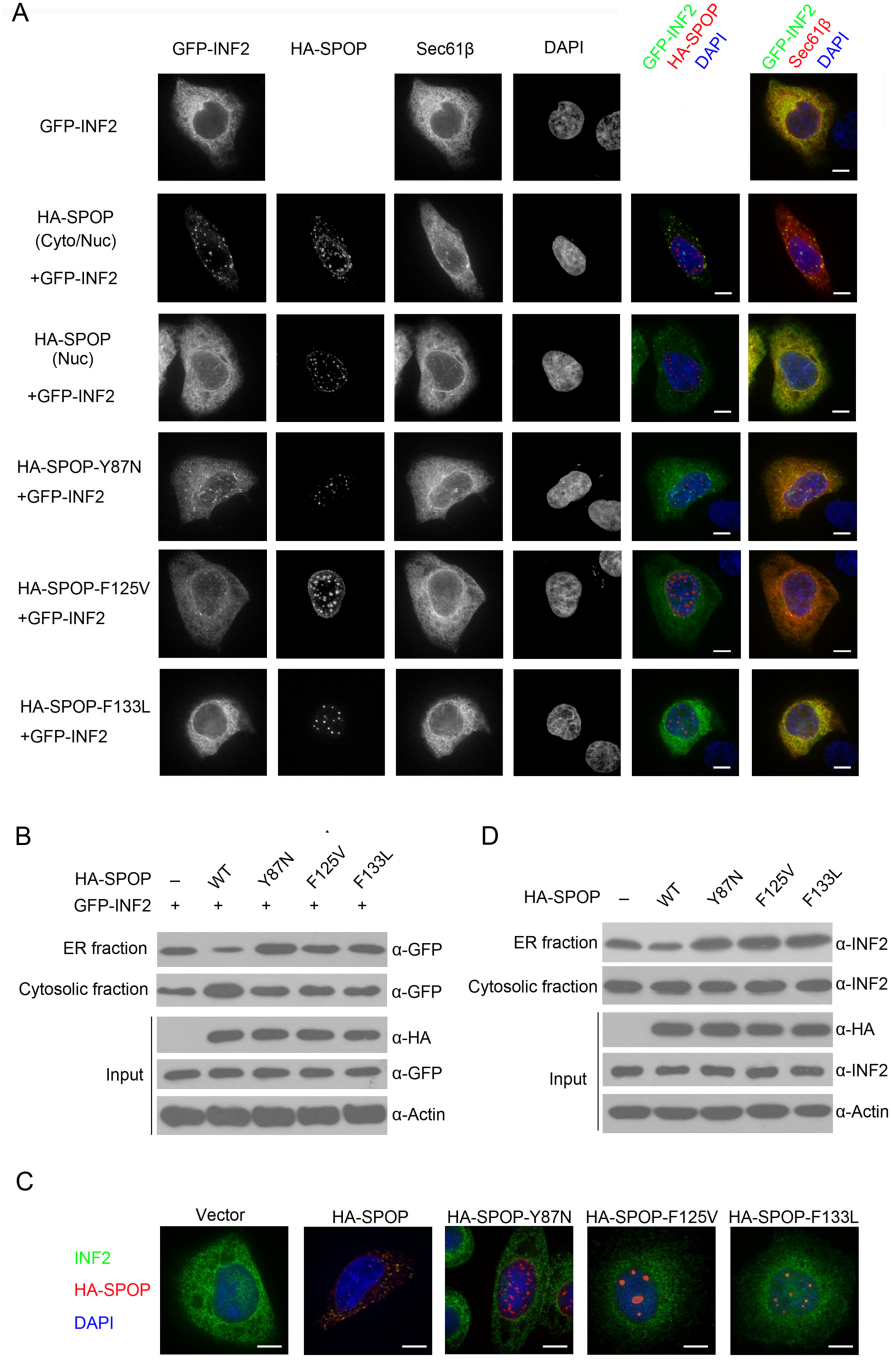
SPOP promotes INF2 disassociation from ER. (A) Representative images of HeLa cells transfected with indicated plasmids, stained with SPOP(HA)and DAPI. Scale bar, 20 μm. (B) HeLa cells were transfected with indicated plasmids. Cytosol and purified ER Fractions were isolated and GFP-INF2 was detected by Western Blot. (C) Representative images of HeLa cells transfected with indicated plasmids, stained with SPOP(HA), INF2 and DAPI. (D) HeLa cells were infected with lentivirus expressing HA-SPOP (WT or mutants) or control. Cytosol and purified ER Fractions were isolated and endogenous INF2 was detected by Western Blot.

INF2 (CAAX isoform) is ER-anchored and INF2-mediated actin assembly is specially triggered at ER-mitochondrial intersections to ensure mitochondrial division [24]. Previous study showed that INF2-CAAX isoform was ER membrane-bound, but a pools of INF2 was cytosolic. [22]. SPOP was originally named as speckle-type POZ protein since ectopically expressed SPOP in COS-7 cells primarily exhibited a discrete speckled pattern in the nucleus [29]. Through quantitative analysis, we found that SPOP was localized exclusively in the nucleus as speckles in approximately 70% cells, but in both the cytoplasm and nucleus in the rest 30% cells, indicating that SPOP shuttles between cytoplasm and nucleus in a proportion of cells (S1 Fig). Thus, we hypothesized that SPOP-INF2 interaction occurs in the cytoplasm and SPOP-mediated atypical ubiquitination may regulate the subcellular localization of INF2. To test this hypothesis, we co-expressed GFP-tagged INF2 (CAAX isoform) and mApple-tagged Sec61β (an ER marker) in cells. We found that these two proteins were perfectly co-localized (Fig 5A), confirming that INF2 CAAX isoform is ER-localized. However, in approximately 30% cells that HA-SPOP was localized in both the cytoplasm and nucleus, INF2 was primarily present as speckles in the cytoplasm and co-localized with SPOP, but not Sec61β (Fig 5A). In contrast, in the rest 70% cells that HA-SPOP was localized exclusively in the nucleus, INF2 was still co-localized Sec61β (Fig 5A). These results suggest that SPOP can inhibit the ER localization of INF2, but this activity strictly depends its cytoplasmic localization. Moreover, deletion of the SBC motif (ΔSBC) or the region containing main ubiquitination sites (ΔInter) in INF2 did not alter its localization in ER (S2 Fig), but SPOP-induced speckle pattern of INF2 in the cytoplasm was not observed (S2 Fig), suggesting that SPOP-INF2 interaction and SPOP-induced INF2 ubiquitination are both required for INF2 localization outside of ER. Next, we investigated the impact of prostate cancer-associated mutants of SPOP on INF2 localization. To this end, we focused on three hotspot mutations Y87N, F125V and F133L. Interestingly, these mutants were exclusively localized as nuclear speckles in nearly 100% cells (S1 Fig), implying that cytoplasmic retention ability of SPOP may be impaired by prostate cancer-associated mutations. Accordingly, we found that ectopic expression of SPOP mutants had no obvious effect on the ER localization of INF2 by immunofluorescence analysis (Fig 5A). We used ER fractionation methods as a second method to corroborate the immunofluorescence analysis. As shown in Fig 5B, overexpression of wild-type SPOP, but not the prostate cancer-associated mutants of SPOP, reduced the protein amounts of GFP-INF2 in ER fractions.

Lastly, we investigated whether SPOP would affect the localization of endogenous INF2. As shown in Fig 5C, in a proportion of SPOP-WT-transfected cells, endogenous INF2 was present in cytoplasmic speckles and co-localized with SPOP, but this effect was not observed in cells expressing SPOP mutants. ER fractionation experiments demonstrated that stably overexpression of wild-type SPOP reduced the protein amounts of endogenous INF2 in ER fractions (Fig 5D). In contrast, overexpression of SPOP mutants moderately increased the protein amounts of endogenous INF2 in ER fractions (Fig 5D), probably those acting through a dominant-negative effect to inhibit endogenous SPOP.

Taken together, our data suggests that wild-type SPOP, but not prostate cancer-associated mutants, can promote INF2 disassociation from ER.

### SPOP antagonizes INF2-mediated mitochondrial fission

**Fig 6:**
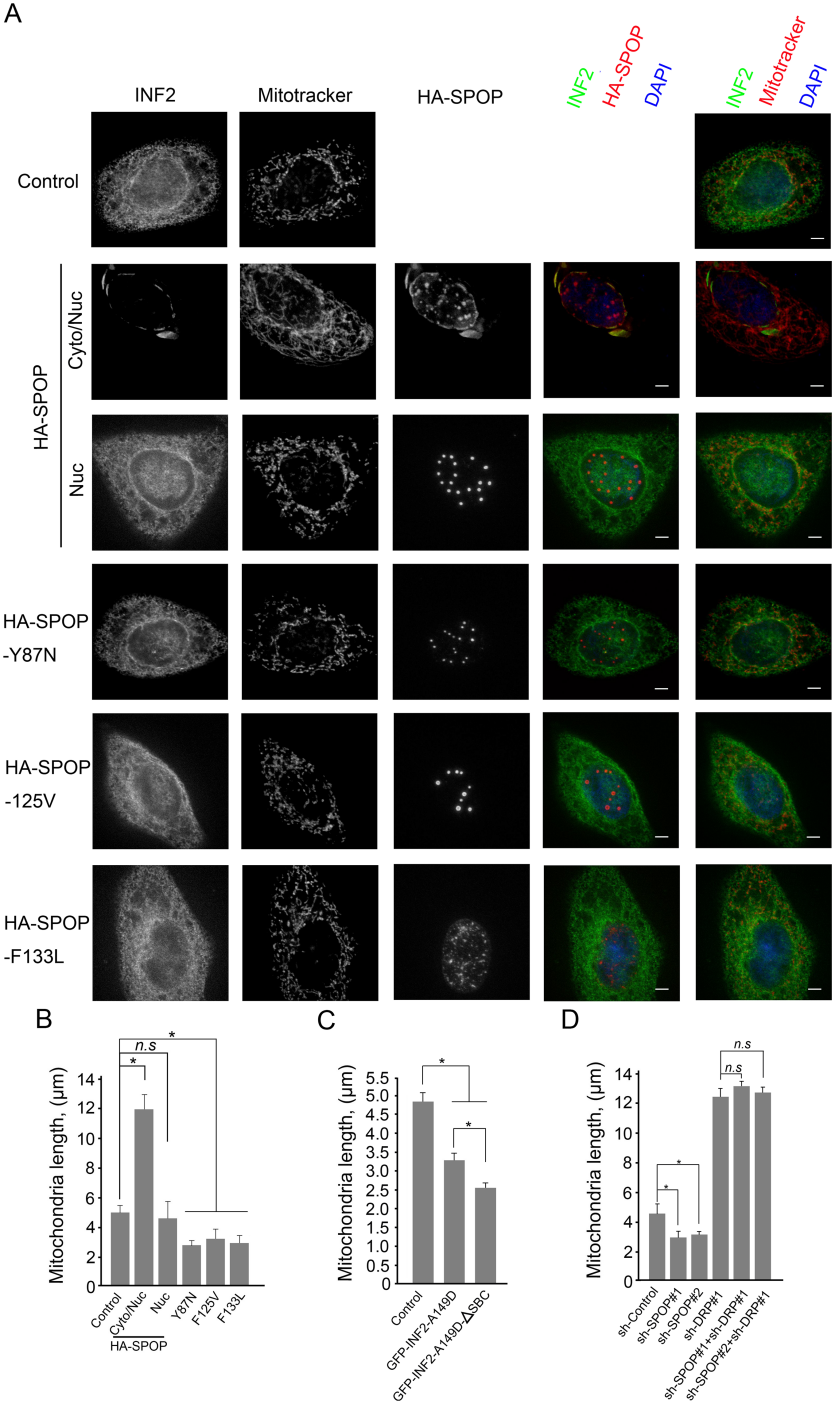
Wild-type SPOP increases, and the prostate cancer-associated SPOP mutants decrease, mitochondria average length. (A) Representative images of DU145 cells infected with lentivirus expressing HA-SPOP (WT or mutants) or control. stained with INF2, SPOP(HA), Mitotracker Red and DAPI. Scale bar, 20 μm. (B) Quantification of mitochondria lengths in (A). n = 40 to 50 cells. Error bars, ± SD for triplicate. (C) DU145 cells were transfected with indicated with indicated plasmids. The mitochondria lengths were quantified similar as (A, B). (D) DU145 cells were infected with lentivirus expressing indicated shRNAs, and the mitochondrial lengths were quantified similar as (A, B).

Considering that actin polymerization between mitochondria and INF2-enriched ER membranes is a critical step in mitochondrial fission [24], we reasoned that SPOP might suppress mitochondrial fission by inhibiting INF2 localization in ER. To test this, DU145 cells were infected with lentivirus expressing wild-type SPOP or prostate cancer-associated SPOP mutants. The mitochondrial morphology was monitored by Mitotracker Red dye. As shown in Fig 6A and 6B, stably overexpression of HA-SPOP resulted in significant increases in mitochondrial average length, accompanying with endogenous INF2 speckles in cytoplasm. However, this effect was only observed in approximately 30% cells that HA-SPOP was localized in both the cytoplasm and nucleus, but not in those cells that HA-SPOP was exclusively localized in nucleus (Fig 6A and 6B). These data suggest that SPOP-mediated suppression of mitochondrial fission strictly depends on its cytoplasmic localization. In contrast, the prostate cancer-associated SPOP mutants (SPOP-Y87N, F125V and F133L) lost the capacity to suppress mitochondrial fission monitored by immunofluorescence (Fig 6A). Statistical analysis showed stably overexpression SPOP mutants even resulted in moderate decreases in mitochondrial average length probably those acting through a dominant-negative effect to inhibit endogenous SPOP (Fig 6B). Previous study reported that the constitutive active mutant INF2-A149D can decreased mitochondrial length [24]. We found that the INF2-A149D-ΔSBC mutant, which can escape from SPOP-mediated ubiquitination, is more potent in decreasing mitochondria average length than INF2-A149D (Fig 6C). Consistent with these findings, depletion of SPOP in DU145 cells resulted in decreases in mitochondria average length (Fig 6D). Moreover, Co-depletion of DRP1 and SPOP by shRNAs reversed the effect of SPOP single depletion on mitochondria size (Fig 6D). Thus, SPOP inactivation-induced mitochondrial fission occurs upstream of DRP1.

Taken together, our data suggest that wild-type SPOP, but not prostate cancer-associated mutants, can suppress INF2-mediated mitochondrial fission.

### SPOP lacking the NLS sequence (SPOP-ΔNLS) is more potent in suppressing mitochondrial fission than SPOP-WT

Our above data indicated that SPOP regulates INF2-mediated mitochondrial fission strictly depends on its cytoplasmic localization, but the nuclear-cytoplasmic shuttling mechanism of SPOP was still poorly understood. It is clear that import of large proteins is generally mediated by nuclear localization signals (NLS), which contain basic amino acids [30]. SPOP contains an evolutionarily conserved NLS sequence at its C-terminus (S3A Fig). We found that SPOP lacking the NLS sequence (SPOP-ΔNLS) accumulated exclusively in the cytoplasm as puncta pattern and perfectly co-localized with GFP-INF2 (S3B Fig). In contrast, two prostate cancer-associated SPOP mutants lacking the NLS sequence (SPOP-F125V-ΔNLS, SPOP-F133L-ΔNLS) accumulated exclusively in the cytoplasm as puncta pattern similar as SPOP-ΔNLS, but these mutants did not co-localize with GFP-INF2, possibly due to impaired interaction with INF2 (S3B Fig). Moreover, SPOP-ΔNLS cannot alter the ER localization of INF2-ΔSBC and INF2-ΔInter mutants (S3C Fig), suggesting that SPOP-INF2 interaction and SPOP-induced INF2 ubiquitination are required for INF2 localization outside of ER. Proteins containing classic NLS are known to be transported into the nucleus by forming complexes with shuttling carriers, such as Karyopherin-alpha and-beta (KPNA and KPNB) [30]. Our yeast two-hybrid screen identified several clones corresponding to KPNA5 (importin subunit alpha-6). Indeed, deletion of the NLS sequence totally abolished the interaction between SPOP(WT, F125V, F133L) and overexpressed or endogenous KPNA5 (S3D Fig), suggesting that KPNA5 might participate in nuclear transport of wild-type and prostate cancer-associated SPOP mutants.

**Fig 7:**
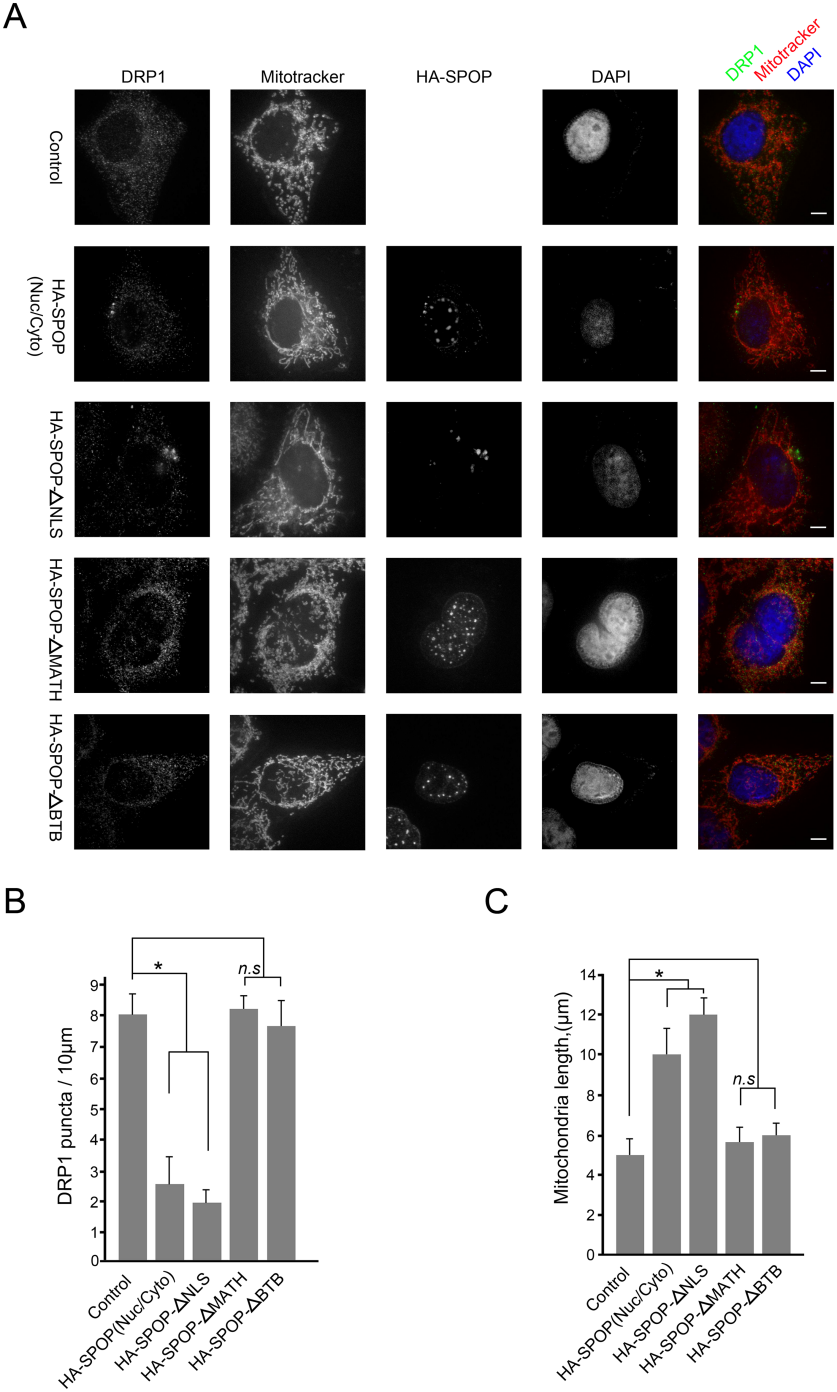
SPOP-ΔNLS mutant is more potent in suppressing mitochondrial fission than SPOP-WT. (A) Representative images of DU145 cells infected with lentivirus expressing HA-SPOP (WT or mutants) or control, stained with DRP1, SPOP(HA), Mitotracker Red and DAPI. (B) Quantification of DRP1 puncta per mitochondrial length in (A). (C) Quantification of mitochondria lengths in (A).

Not surprisingly, we found that SPOP-ΔNLS was able to immunoprecipitate more endogenous INF2 than SPOP-WT(S3E Fig), and SPOP-ΔNLS was more effective to promote INF2 ubiquitination than SPOP-WT(S3F Fig). It has been reported that INF2 promotes mitochondrial fission controls mitochondrial assembly of DRP1 [24]. DRP1 localized to cytoplasm and to mitochondrially associated puncta in cells. Depletion of INF2 reduced mitochondrially associated puncta, in addition to causing mitochondrial elongation[24]. We observed that SPOP-ΔNLS overexpression reduced DRP1 puncta associated with mitochondria and increased mitochondria length more efficient than SPOP-WT (Fig 7A, 7B and 7C). The levels of DRP1 in purified mitochondrial fractions from SPOP-ΔNLS overexpressing cells were also lower than those from SPOP-WT overexpressing cells (S3G Fig). In contrast, overexpression of SPOP-ΔBTB or ΔMATH mutant had no impact on DRP1 puncta, and mitochondria length (Fig 7A, 7B and 7C). It is not surprising since INF2 protein cannot be polyubiquitinated by SPOP-ΔBTB or ΔMATH.

Taken together, our data confirmed that cytoplasmic retention of SPOP is required for its regulation of mitochondrial fission.

### INF2-mediated mitochondrial fission is involved in SPOP inactivation-promoted cell migration and invasion

**Fig 8:**
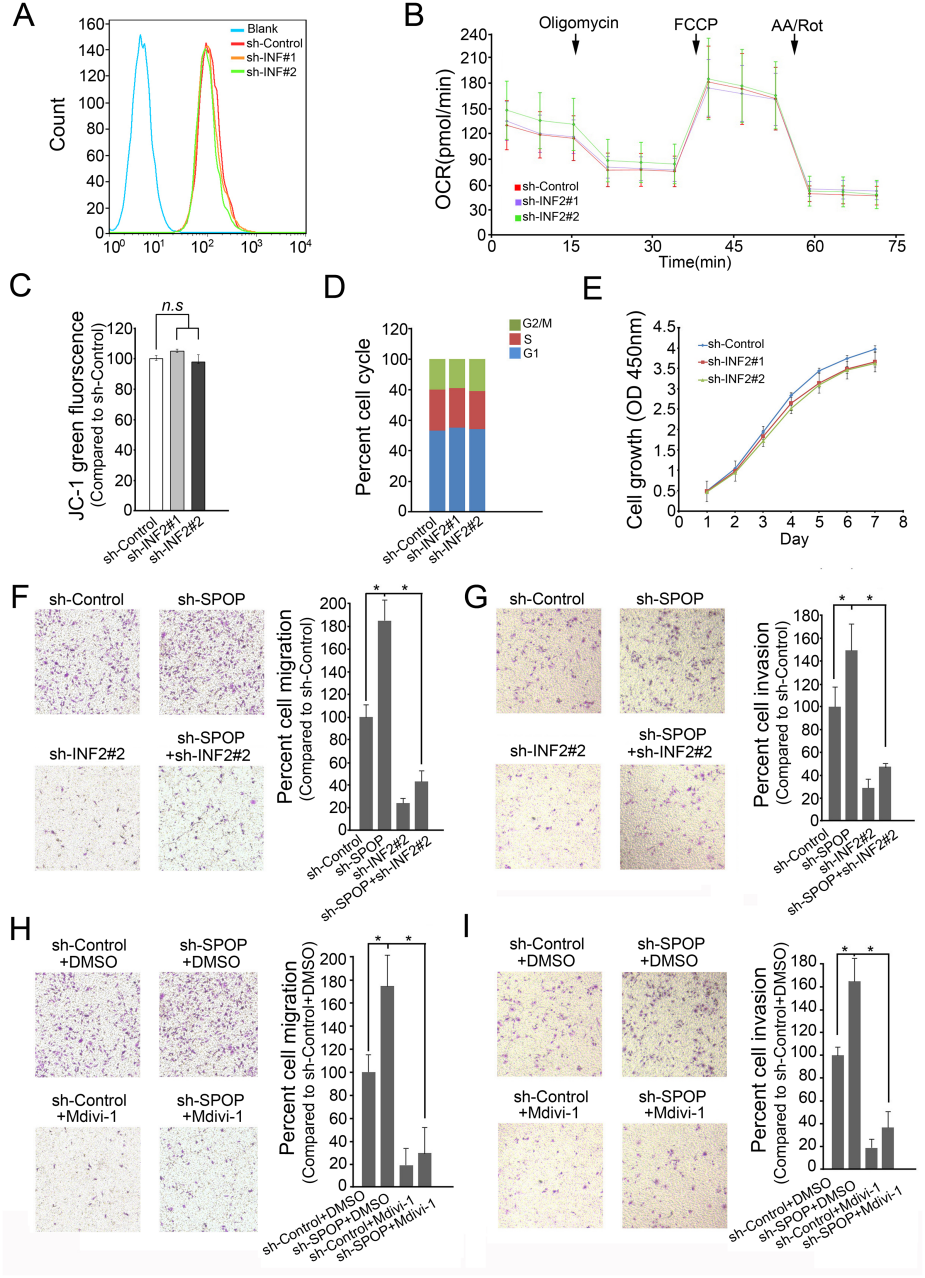
SPOP suppresses cell invasion and migration partially dependent on INF2. (A) MitoSOX Red was added to DU145 cells expressing shRNAs targeting INF2 or scramble control and fluorescence was measured by flow cytometry. (B) Oxygen consumption rate was measured using an XF24 extracellular flux analyzer in DU145 cells expressing shRNA targeting INF2 or scramble control. Oligomycin, FCCP, Rotenone and Antimycin A were added at the indicated timepoints (arrows). Spare respiratory capacity is measured as the difference between basal oxygen consumption rate and the FCCP uncoupled oxygen consumption rate. (C) JC-1 fluorescent dye was added to DU145 cells expressing shRNA targeting INF2 or scramble control. For quantification, the green fluorescence intensity (representing the degree of decreased ΔΨm) was measured by flow cytometry. Data represent three replicates. *n*.*s*, not statistically significant. (D) Cell cycle analysis of DU145 cells infected with lentivirus expressing indicated shRNAs. (E) Cell growth analysis of DU145 cells infected with lentivirus expressing indicated shRNAs. (F) DU145 cells were infected with lentivirus expressing indicated shRNAs. Cell migration assay was shown on the left panel, and the quantitative analysis is shown on the right panel. All data shown are mean values ± SD (error bar) from three replicates. *p < 0.01. (G) DU145 cells were infected with lentivirus expressing indicated shRNAs for cell invasion assay. (H) DU145 cells were infected with lentivirus expressing indicated shRNAs and treated with Mdivi-1 (1 μM)for cell migration assay. (I) DU145 cells were infected with lentivirus expressing indicated shRNAs and treated with Mdivi-1 (1 μM)for cell invasion assay.

To determine the biological importance of SPOP regulation of INF2-mediated mitochondrial fission, we first used two independent shRNAs (#1 targets total INF2, #2 targets INF2 CAAX isoform only) to knock down INF2 expression. Consistent with previous studies, INF2 depletion in LNCaP or DU145 cells resulted in a significant increase in mitochondrial average length (S4 Fig). However, this change was not associated with major change in mitochondrial function, as the basal mitochondrial reactive oxygen species (ROS) production (Fig 8A), oxygen consumption rate (OCR) (Fig 8B), and membrane potential (Fig 8C) were not significantly altered following INF2 depletion. Moreover, we found that INF2 depletion marginally affected the cell cycle progression (Fig 8D) or overall cell growth (Fig 8E). These results led us to explore other cancer cell phenotypes affected by INF2 depletion.

Recently, emerging evidence supports a role for mitochondrial dynamics in tumor cell migration and invasion in various cancer models [16–20]. Indeed, we found that depletion of INF2 in DU145 cells markedly decreased cell migration and invasion (Fig 8F and 8G). In contrast, depletion of SPOP enhanced cell migration and invasion (Fig 8F and 8G). More importantly, co-depletion of SPOP and INF2 reduced cell migration and invasion compared with depletion of SPOP only (Fig 8F and 8G). Similar results were obtained when we used SPOP-F133L mutant overexpression to replace knockdown of SPOP by shRNA (S5A and S5B Fig). Previous studies demonstrated that INF2 functions upstream of DRP1[24]. We found that treatment with DU145 cells with DRP1 selective inhibitor Mdivi-1 or knockdown of DRP1 significantly reduced SPOP depletion-enhanced cell migration and invasion (Fig 8H and 8I; S5C and S5D Fig). Similar effects were observed in another prostate cancer cells LNCaP (S6 Fig). Together, our data suggests that SPOP suppresses prostate cell migration and invasion, at least in part, by regulating INF2-mediated mitochondrial fission.

## Discussion

**Fig 9:**
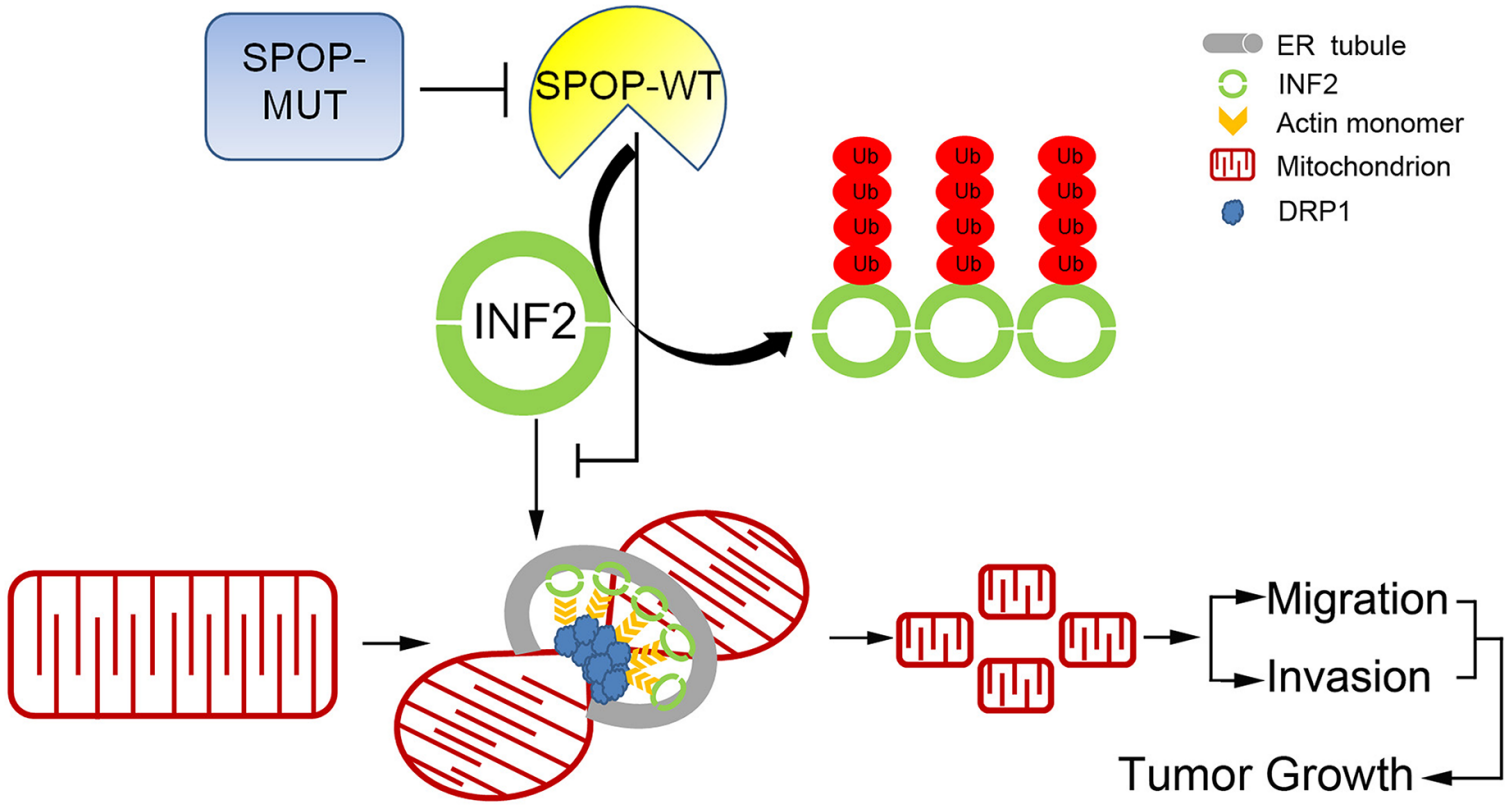
A proposed model how SPOP is a regulator of mitochondrial fission and dysregulated by prostate cancer-associated mutations.

Although SPOP mutation is now recognized as a distinct molecular feature in a subtype of prostate cancer, the underlying mechanisms remain poorly understood [4]. Previous studies showed that SPOP inactivation increased cell proliferation primarily in AR-positive prostate cancer cells, but increased prostate cell migration and invasion in an AR-independent manner [2,9,10]. These effects were partly dependent on stabilization of SPOP substrates such as AR and ERG [9,10]. ERG up-regulation leads to transactivation of its target genes, including ADAMTS1, CXCR4, OPN and MMP9, all of which play important roles in promoting cell migration and invasion [9,10]. In this study, we revealed that the ER-localized isoform of the INF2 is ubiquitinated and regulated by SPOP. SPOP inactivation-induced prostate cancer cell migration and invasion is partly mediated by INF2 and mitochondrial fission (Fig 9). In the past few years, there is accumulating evidence that mitochondrial fission and fusion play active roles in regulation of cell movement, migration and invasion [14,31]. For example, there are higher levels of DRP1 and less Mfn1 (a GTPase for mitochondrial fusion) in the metastatic breast cancer cells compared with non-metastatic breast cancer cells.^18^ Silencing DRP1 or overexpression of Mfn1 results in mitochondrial fusion, and significantly suppresses migration and invasion abilities of breast cancer cells [18]. Similar effect has been detected in glioblastoma and lung or thyroid cancer[17,19,20]. The possible mechanism for mitochondrial fission-enhanced cell movement is that mitochondria are usually trafficked to sites of high-energy demand, and in migrating cells, mitochondria are more frequently located at their leading edge where demands high energy [14,32]. Our study, for the first time, links prostate cancer-associated SPOP mutations to mitochondrial dynamics-related cell migration and invasion. Interestingly, a recent study demonstrated that aberrant activation of MAPK signaling by K-Ras (G12V) mutation in pancreatic cancer activates DRP1 via ERK-mediated phosphorylation, and DRP1-meditated mitochondrial fission is crucial for Ras-driven transformation [33]. Similarly, BRAF (V600E), the most common mutation in melanoma, correlates with DRP1 phosphorylation in melanoma tumor tissues, whereas MAPK inhibition reverses DRP1-mediated mitochondrial fission, and sensitizes cells to mitochondrial-targeting drugs [34]. Therefore, cancer-associated mutations may promote mitochondrial fission through multiple signaling pathways in different tumors. It also should be noted that SPOP can ubiquitinate the cytoplasmic INF2 non-CAAX isoform similar as the ER-localized INF2 CAAX isoform (Fig 2F). A recent study revealed that a proportion of cytoplasmic INF2 was localized in focal adhesion (FA) and the protruding edge of migrating cells [35]. We cannot rule out the possibility that SPOP-mediated ubiquitination of INF2 non-CAAX isoform also affects cell migration and invasion.

Ubiquitination has critical functions in nearly all aspects of biological processes. Although ubiquitination is traditionally thought to only target proteins for degradation, recent studies suggest additional roles of ubiquitination in nonproteolytic functions involved in protein function regulation [36]. It is well known that K48-linked polyUb ubiquitin chains are sufficient to target substrates to the 26S proteasome for degradation and that K63-linked polyUb ubiquitin chains have been demonstrated to regulate a variety of nonproteolytic cellular functions, though the roles of other atypical ubiquitin linkages through M1, K6, K11, K27, K29 or K33 or mixed linkages within the same chain remain poorly understood [36]. Previous studies demonstrated that the mitochondrial ubiquitin ligase MITOL regulates mitochondrial-ER membrane bridges through K63-linked ubiquitination of mitochondrial Mfn2 (a GTPase for mitochondrial fission), suggesting that atypical ubiquitination plays roles in mitochondrial dynamics [37]. In this study, we demonstrated SPOP catalyzes synthesis of mixed-linkage polyUb chains (K27, K29, K33, K48 and K63) on INF2, which does not trigger INF2 degradation. Instead, these forms of ubiquitination cause INF2 dissociation from ER and impair its ability to promote mitochondrial fission. Until now, the majorities of known SPOP substrates are ubiquitinated and degraded by SPOP. But a previous study showed that SPOP is able to ubiquitinate the PcG protein BMI1 and the histone variant MacroH2A. These ubiquitinations do not affect the overall stability of BMI1 or MacroH2A, but facilitates PcG-mediated transcriptional repression and deposition of MacroH2A during stable X chromosome inactivation process [38]. These data and others reinforce a notion that SPOP can promote both degradative or non-degradative ubiquitination towards different substrates. Moreover, it is also possible that unknown deubiquitinase(s) might exist to recycle INF2 from cytoplasmic speckles to ER.

Another interesting aspect of our work that needs further investigation is the potential molecular mechanism that accounts for nuclear-cytoplasmic shuttling of SPOP. One study demonstrated that hypoxia condition promotes SPOP cytoplasmic accumulation in clear cell renal cell carcinoma (ccRCC) cells [39]. However, our preliminary results found that hypoxia treatment did not affect SPOP localization at least, in prostate cancer cells (S7 Fig). Considering that SPOP-mediated suppression of mitochondrial fission is strictly dependent on its cytoplasmic localization, elucidation the molecular mechanisms of cytoplasmic accumulation of SPOP is an important direction to pursue in the future. Moreover, three prostate cancer-associated SPOP mutants (Y87N, F125V and F133L) nearly lost their cytoplasmic localization compared with wild-type SPOP. It is possible that these mutations impair the capacity of SPOP to interact with proteins which facilitate cytoplasmic retention of SPOP. Our results showed that deletion of the NLS sequence forced prostate cancer-associated SPOP mutants to localize in cytosol as puncta, but these mutants cannot alter the ER localization of INF2 like SPOP-WT (Fig 7B). So it is possible that the direct interaction with some cytoplasmic substrates of SPOP, including but not limited to INF2, may cause a pool of SPOP to accumulate in cytosol by blocking access to Importin proteins. Prostate cancer-associated SPOP mutants lost the capacity to interact with its cytoplasmic binding partner, and localized exclusively in the nucleus. Taken together, our data suggest SPOP might exert its tumor-suppressive roles both in nucleus and cytoplasm.

## Materials and methods

### Cell culture

293T, HeLa cells and prostate cancer cell lines (LNCaP, DU145, PC-3) were obtained from the American Type Culture Collection (ATCC). 293T and HeLa cells were maintained in DMEM with 10% (v/v) FBS. LNCaP and DU145 cells were maintained in DMEM with 10% (v/v) FBS. All cells were grown at 37°C with 5% CO_2_.

### Plasmids constructions

Expression vectors for SPOP-WT or mutants are described previously. FLAG-INF2-CAAX was obtained from Dr. Miguel Angel Alonso (Universidad Autónoma de Madrid). INF2 mutants were generated by KOD-Plus-Mutagenesis Kit (TOYOBO) following the manufacturer’s instructions.

### Subcellular fractionation

For WB detection of ER-localized INF2 from HeLa cells, the microsomal fraction from approximately 5×10^6^ HeLa cells was prepared using an ER extraction kit (ER0100, Sigma-Aldrich). The mitochondrial fraction was prepared using a Mitochondria Isolation Kit (MitoISO1, Sigma-Aldrich).

### Lentiviral preparation, viral infection, and stable cell generation

The pLKO.3G GFP-shRNA plasmids were purchased from Addgene. The shRNA sequence of sh-SPOP#1: 5’-GGAGAACGCUGCAGAAAUU-3’; sh-SPOP#2: 5’-ATAAGTCCAATAACGACAGGC-3’; shINF2-#1: 5’- CCCUCUGUGGUCAACUACU-3’; shINF2-#2 (target to CAAX isoform only): 5’-ACAAAGAAACTGTGTGTGTGA-3’;^23^ shDRP1: 5’-GCCAGCUAGAUAUUAACAACAAGAA-3’. shControl: 5’- ACAGACUUCGGAGUACCUG-3’. Viruses were collected from the medium 48 hr after transfection. For knockdown experiments, cells were infected with the collected viruses over 48 hr in the presence of polybrene, followed by GFP sorting for 3–4 days. pTsin- lentivirus vectors were used for overexpression of HA(FLAG)-SPOP-WT or mutants.

### Antibodies and chemicals

The following antibodies were used: SPOP (ab137537; Abcam), SPOP (16750-1-AP; proteintech), INF2 (20466-1-AP; proteintech), AR (SC-816; Santa Cruz), DEK (16448-1-AP, Proteintech), IQGAP1(ab133490; Abcam), DRP1(8570S; CST), KPNA5(A7731; Abcam), COX4 (Abcam; ab14744), Ubiquitin (6652–1; epitomics), Myc (9E10; Sigma), FLAG (M2; Sigma), HA (MM5-101R; Convance), Actin (AC-74; Sigma). Mdivi-1 was purchased from Selleckchem. MitoSOX Red dye was purchased from Invitrogen.

### Mass spectrometry analysis of ubiquitin attachment sites

Ubiquitinated INF2 was prepared by transfecting FALG-INF2, HA-Ub and Myc-SPOP in 293T cells (5x100 mm dish). After 48 hr, the cells were lysed in RIPA buffer and the transfected INF2 was immunopurified from cell lysates with anti-Flag M2 agarose beads (Sigma) before being resolved by 7.5% SDS-PAGE. After Coomassie blue staining, the band corresponding to ubiquitinated INF2 was excised. The liquid chromatography tandem mass spectrometry analysis was carried out at the Proteomics Center of our institute.

### Cell cycle analysis

For cell cycle analysis, cells were washed 48 h post-treatment with PBS and fixed in 70% ethanol overnight. The cells were washed again with PBS, stained with propidium iodide and analyzed by flow cytometry.

### Cell proliferation assay

Cell proliferation rate was determined using Cell Counting Kit-8 (CCK-8) according to the manufacturer’s protocol (Dojindo Laboratories, Japan). Briefly, the cells were seeded onto 96-well plates at a density of 1,000 cells per well. During a 2 to 8-d culture periods, 10 μl of the CCK-8 solution was added to cell culture, and incubated for 2 hr. The resulting color was assayed at 450 nm using a microplate absorbance reader (Bio-Rad). Each assay was carried out in triplicate.

### Migration and invasion assays

Cell migration and invasion were determined by Transwell (Costar) migration and invasion assays. LNCaP cells were precultured in serum-free medium for 48 hr. For migration assay, 3x10^4^ cells were seeded in serum-free medium in the upper chamber, and the lower chamber was filled with RPMI1640 containing 5% FBS. After 48 h, the non-migrating cells on the upper chambers were carefully removed with a cotton swab, and migrated cells underside of the filter stained and counted in nine different fields. Matrigel invasion assays were performed using Transwell inserts (Costar) coated with Matrigel (BD Biosciences)/fibronectin ((BD Biosciences).

### OCR assay

OCR was measured using a Seahorse XF24 Extracellular Flux Analyzer with the XF Cell Mito Stress Test Kit. Cells were seeded at 8 x 10^4^ cells per well in 100μl DMEM containing 10% FBS and allowed to attach for 2 hr. 150μl DMEM-10% FBS was added per well and cells incubated overnight in 5% CO_2_ humidified incubator. Prior to assay run, cells were changed into assay media, unbuffered DMEM pH 7.4 and subjected to sequential injections of Oligomycin (1 μM), FCCP (0.3 μM), rotenone (1 μM) and antimycin A (0.75 μM). Spare respiratory capacity was calculated by dividing the OCR response to FCCP by the basal respiration, having subtracted the non-mitochondrial respiration previously. All values were normalized to cell number per wells setup in parallel.

### Determination of mitochondrial membrane potential (ΔΨm) and mtROS

Cells were seeded for 24hr and treated as indicated. TMRE (50 nM) or MitoSOX Red (5 μM) was added to the media, and the plates were incubated at 37°C in the dark for 30 min. Then cells were trypsinized and analyzed by flow cytometry.

### Immunofluorescence and confocal microscopy

For immunofluorescence, cells were plated on chamber slides, fixed with 4% paraformaldehyde at room temperature for 30 min. After washing with PBS, cells were permeabilized with 0.1% Triton X-100 in PBS for 15 min. Cells were then washed with PBS, blocked with 0.5% BSA in PBS for 1hr, and incubated with primary antibodies in PBS for at 4°C for overnight. After washing with PBS, fluorescence-labelled secondary antibodies were applied and DAPI was counterstained for 1hr at room temperature. Cells were visualized and imaged using a confocal microscope (LSM710, Zeiss). The analytic method of mitochondrial length was described previously.^23^

## Supporting information

S1 Fig The subcellular localization of wild-type SPOP or prostate cancer-associated SPOP mutants. (A) Representative images of HeLa cells transfected with indicated plasmids, stained with SPOP(HA) and DAPI. Scale bar, 20 μm. (B) The relative Nuc/Cyto localization was quantified. n = 80 to 100 cells. Error bars, ± SD for triplicate.
(TIF)

S2 Fig SPOP promotes INF2 disassociation from ER (related to Fig 5). (A) Representative images of HeLa cells transfected with indicated plasmids, stained with SPOP(HA) and DAPI. Scale bar, 20 μm. (B) HeLa cells were co-transfected with HA-SPOP and GFP-INF2 mutant (ΔSBC or ΔInter). Cytosol and purified ER Fractions were isolated and ER-localized GFP-INF2 was detected by Western Blot.
(TIF)

S3 Fig SPOP-ΔNLS mutant is constitutively localized in cytosol as puncta and more potent in suppressing mitochondrial fission than SPOP-WT (related to Fig 5). (A) Diagram showing C-terminal NLS sequence in SPOP, as well as the sequence alignment of NLS sequence among different species to illustrate that this motif is evolutionarily conserved. (B) Representative images of DU145 cells transfected with indicated plasmids, stained with SPOP(HA) and DAPI. Scale bar, 20 μm. (C) Representative images of DU145 cells transfected with indicated plasmids, stained with SPOP(HA) and DAPI. (D) The NLS motif is required for SPOP interaction with KPNA5. (*left*) Western blot of WCL and co-IP samples of anti-FLAG antibody obtained from 293T cells transfected with indicated plasmids. (*right*) Western blot of WCL and co-IP samples of anti-FLAG antibody obtained from 293T cells transfected with indicated plasmids. (E) Western blot of WCL and co-IP samples of anti-FLAG antibody obtained from DU145 cells infected with lentivirus expressing FLAG-SPOP or control. (F) Western blot of the products of *in vivo* ubiquitination assay performed using cell lysates from 293T cells transfected with indicated plasmids. (G) DU145 cells infected with lentivirus expressing HA-SPOP-WT, or ΔNLS mutants or control. Cytosol and purified mitochondrial fractions were isolated and DRP1 was detected by Western Blot.
(TIF)

S4 Fig Knockdown of INF2 increases mitochondrial average length. (A, B) LNCaP or DU145 cells were infected with lentivirus expressing indicated shRNAs and stained with Mitotracker Red and DAPI, and the mitochondrial average lengths were measured (B). n = 35 to 40 cells. Error bars, ± SD for triplicate. Scale bar, 20 μm.
(TIF)

S5 Fig SPOP suppresses cell invasion and migration partially dependent on INF2 (related to Fig 8). (A, B) DU145 cells were infected with lentivirus expressing HA-SPOP-F133L or control, then the stable cell lines were subsequent infected with lentivirus expressing sh-INF2 or sh-control. Cell migration assay (A) and invasion assay (B) were performed. *p < 0.01. (C, D) DU145 cells were infected with lentivirus expressing indicated shRNAs for cell migration and invasion assay.
(TIF)

S6 Fig SPOP suppresses cell invasion and migration partially dependent on INF2 (LNCaP cells). (A) MitoSOX Red was added to LNCaP cells expressing shRNA targeting INF2 or scramble control and fluorescence was measured by flow cytometry. (B) Oxygen consumption rate was measured using an XF24 extracellular flux analyzer in LNCaP cells expressing shRNA targeting INF2 or scramble control. Oligomycin, FCCP, Rotenone and Antimycin A were added at the indicated timepoints (arrows). Spare respiratory capacity is measured as the difference between basal oxygen consumption rate and the FCCP uncoupled oxygen consumption rate. (C) JC-1 fluorescent dye was added to LNCaP cells expressing shRNA targeting INF2 or scramble control. For quantification, the green fluorescence intensity (representing the degree of decreased ΔΨm) was measured by flow cytometry. Data represent three replicates. *n*.*s*, not statistically significant. (D) Cell cycle analysis of LNCaP cells infected with lentivirus expressing indicated shRNAs. (E) Cell growth analysis of LNCaP cells infected with lentivirus expressing indicated shRNAs. (F) LNCaP cells were infected with lentivirus expressing indicated shRNAs. Cell migration assay was shown on the left panel, and the quantitative analysis is shown on the right panel. All data shown are mean values ± SD (error bar) from three replicates. *p < 0.01 from triplicate. (G) LNCaP cells were infected with lentivirus expressing indicated shRNAs for cell invasion assay. (H) LNCaP cells were infected with lentivirus expressing indicated shRNAs and treated with Mdivi-1 (1 μM) for cell migration assay. (i) LNCaP cells were infected with lentivirus expressing indicated shRNAs and treated with Mdivi-1 (1 μM) for cell invasion assay.
(TIF)

S7 Fig The subcellular localization of HA-SPOP in LNCaP cells under hypoxic conditions. LNCaP cells infected with lentivirus expressing HA-SPOP, and treated with DMSO or CoCl_2_(100 μM) for 24hr. The relative Nuc/Cyto localization was quantified. n = 80 to 100 cells. Error bars, ± SD for triplicate.
(TIF)
