# Supplementary figures and images for: Dysregulation of INF2-mediated mitochondrial fission in SPOP-mutated prostate cancer
Source: PLoS Genet. 2017 Apr 27;13(4):e1006748. doi: 10.1371/journal.pgen.1006748 (PMC5426793; doi:10.1371/journal.pgen.1006748)

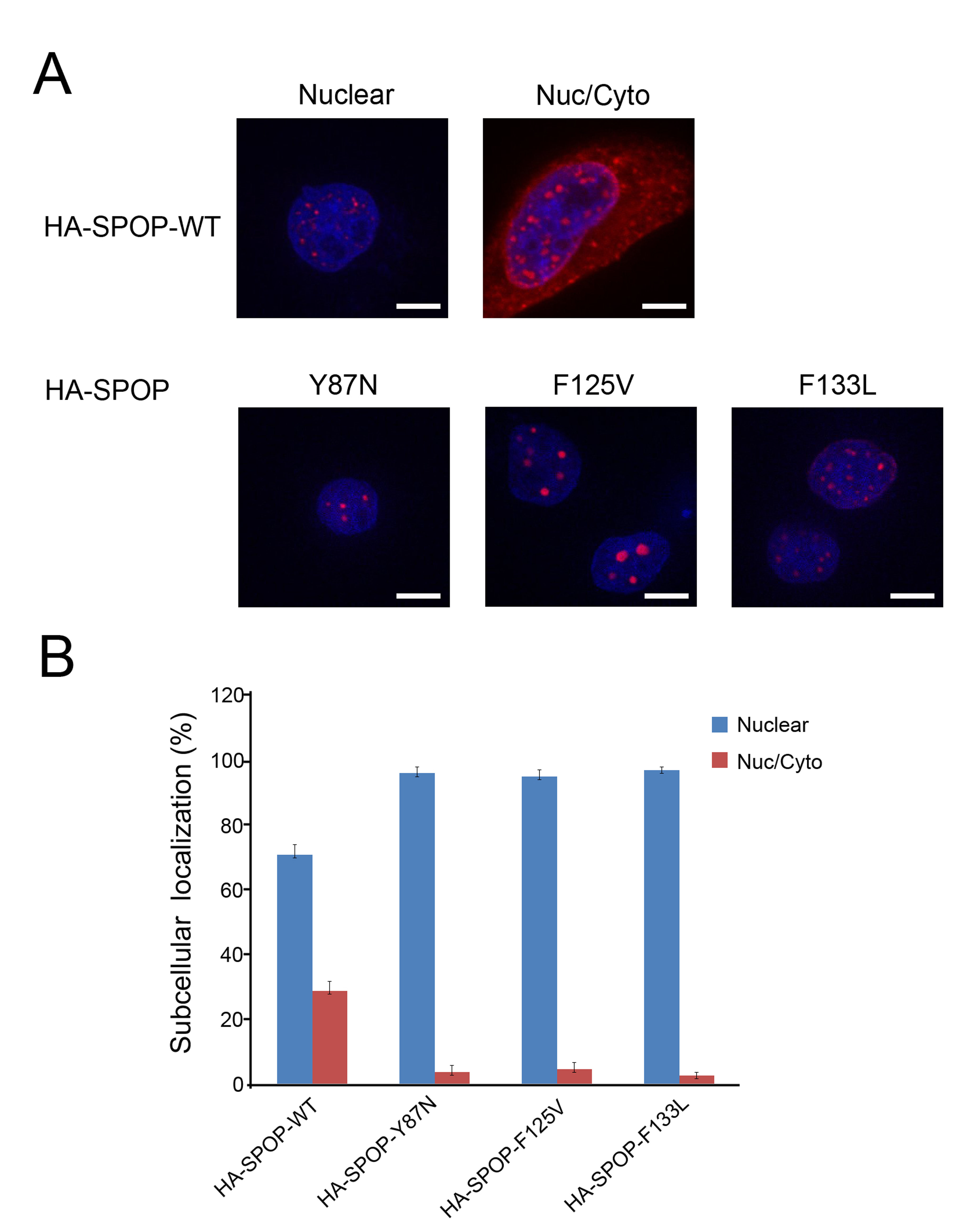

Supplement: S1 Fig — (A) Representative images of HeLa cells transfected with indicated plasmids, stained with SPOP(HA) and DAPI. Scale bar, 20 μm. (B) The relative Nuc/Cyto localization was quantified. n = 80 to 100 cells. Error bars, ± SD for triplicate. (TIF) [file pgen.1006748.s001.tif]

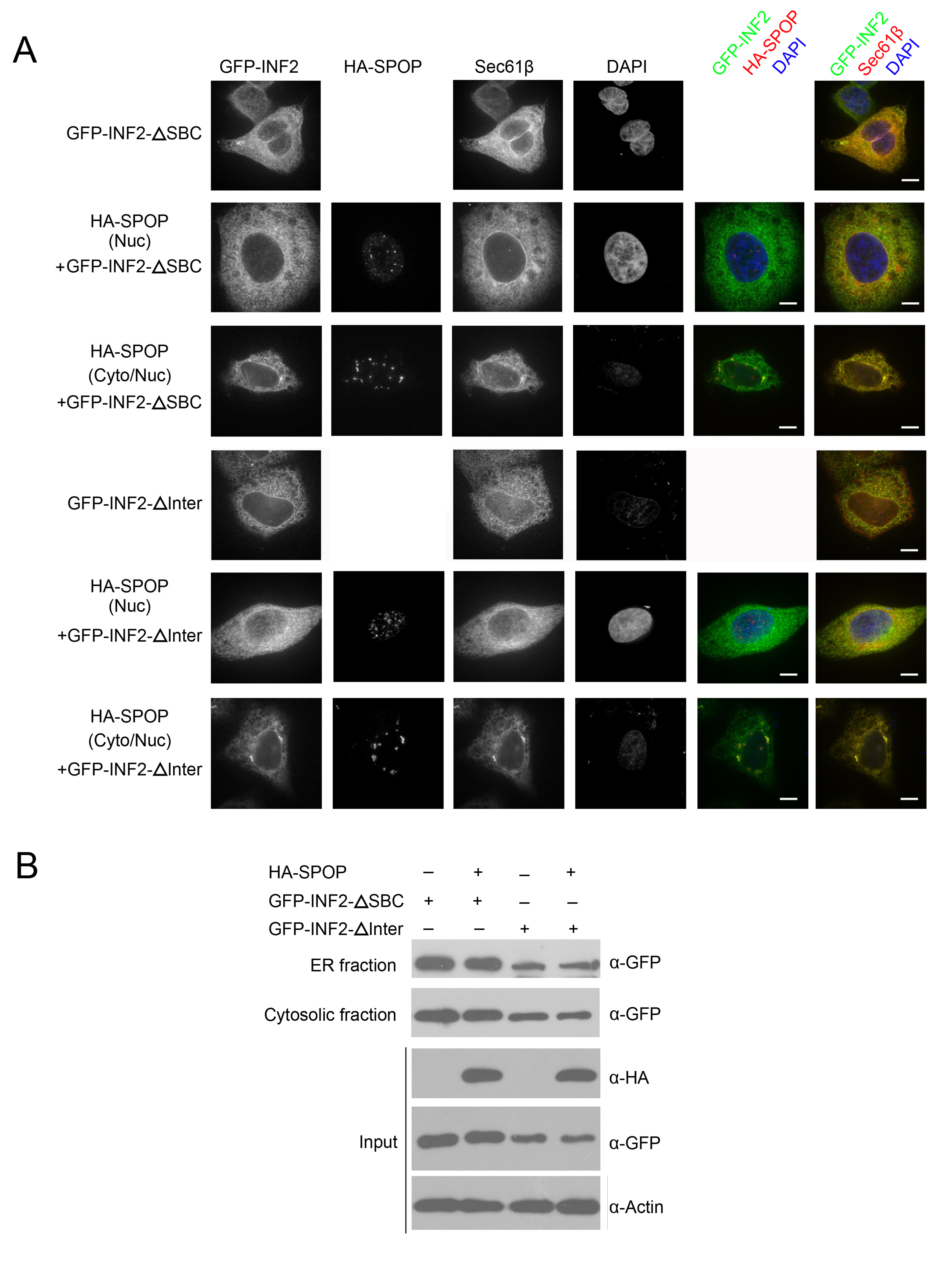

Supplement: S2 Fig — (A) Representative images of HeLa cells transfected with indicated plasmids, stained with SPOP(HA) and DAPI. Scale bar, 20 μm. (B) HeLa cells were co-transfected with HA-SPOP and GFP-INF2 mutant (ΔSBC or ΔInter). Cytosol and purified ER Fractions were isolated and ER-localized GFP-INF2 was detected by Western Blot. (TIF) [file pgen.1006748.s002.tif]

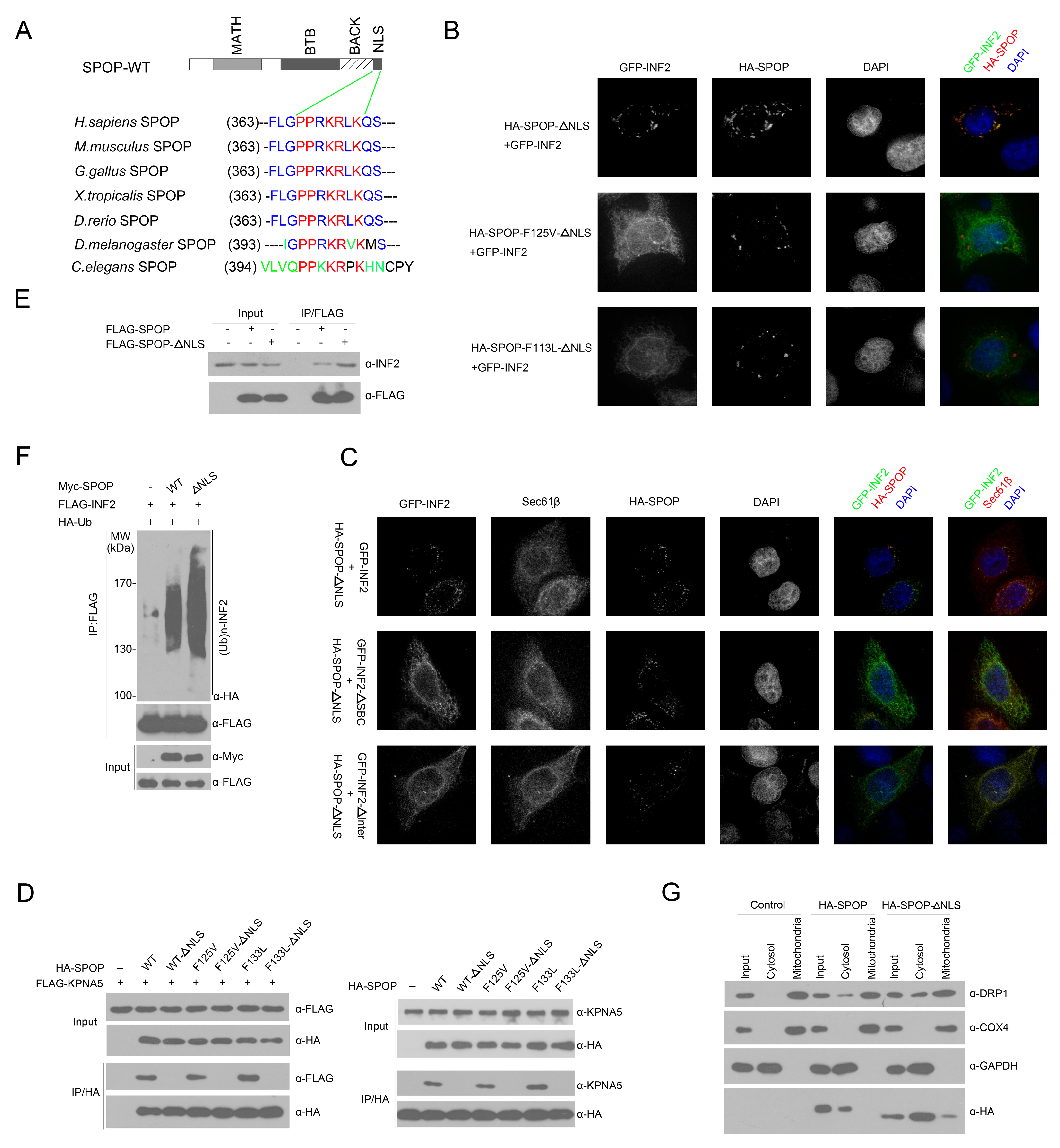

Supplement: S3 Fig — (A) Diagram showing C-terminal NLS sequence in SPOP, as well as the sequence alignment of NLS sequence among different species to illustrate that this motif is evolutionarily conserved. (B) Representative images of DU145 cells transfected with indicated plasmids, stained with SPOP(HA) and DAPI. Scale bar, 20 μm. (C) Representative images of DU145 cells transfected with indicated plasmids, stained with SPOP(HA) and DAPI. (D) The NLS motif is required for SPOP interaction with KPNA5. (left) Western blot of WCL and co-IP samples of anti-FLAG antibody obtained from 293T cells transfected with indicated plasmids. (right) Western blot of WCL and co-IP samples of anti-FLAG antibody obtained from 293T cells transfected with indicated plasmids. (E) Western blot of WCL and co-IP samples of anti-FLAG antibody obtained from DU145 cells infected with lentivirus expressing FLAG-SPOP or control. (F) Western blot of the products of in vivo ubiquitination assay performed using cell lysates from 293T cells transfected with indicated plasmids. (G) DU145 cells infected with lentivirus expressing HA-SPOP-WT, or ΔNLS mutants or control. Cytosol and purified mitochondrial fractions were isolated and DRP1 was detected by Western Blot. (TIF) [file pgen.1006748.s003.tif]

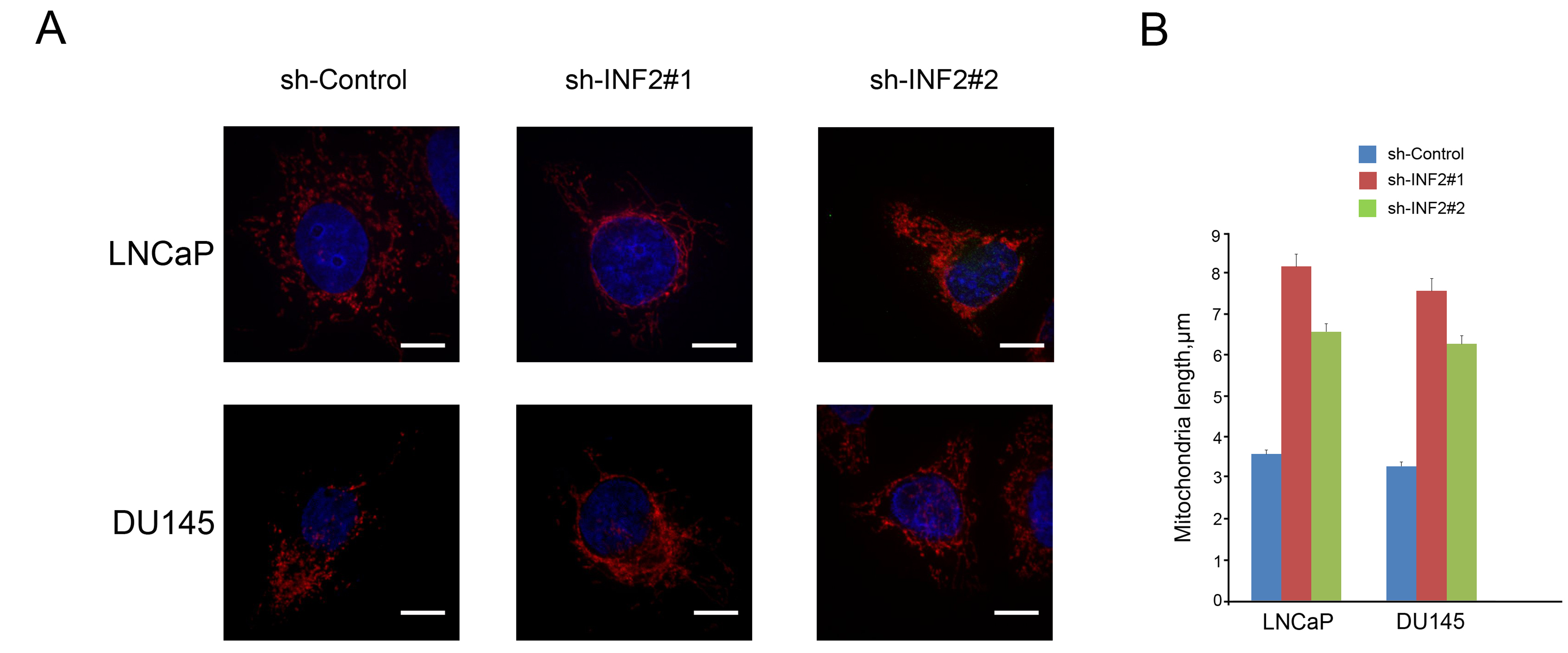

Supplement: S4 Fig — (A, B) LNCaP or DU145 cells were infected with lentivirus expressing indicated shRNAs and stained with Mitotracker Red and DAPI, and the mitochondrial average lengths were measured (B). n = 35 to 40 cells. Error bars, ± SD for triplicate. Scale bar, 20 μm. (TIF) [file pgen.1006748.s004.tif]

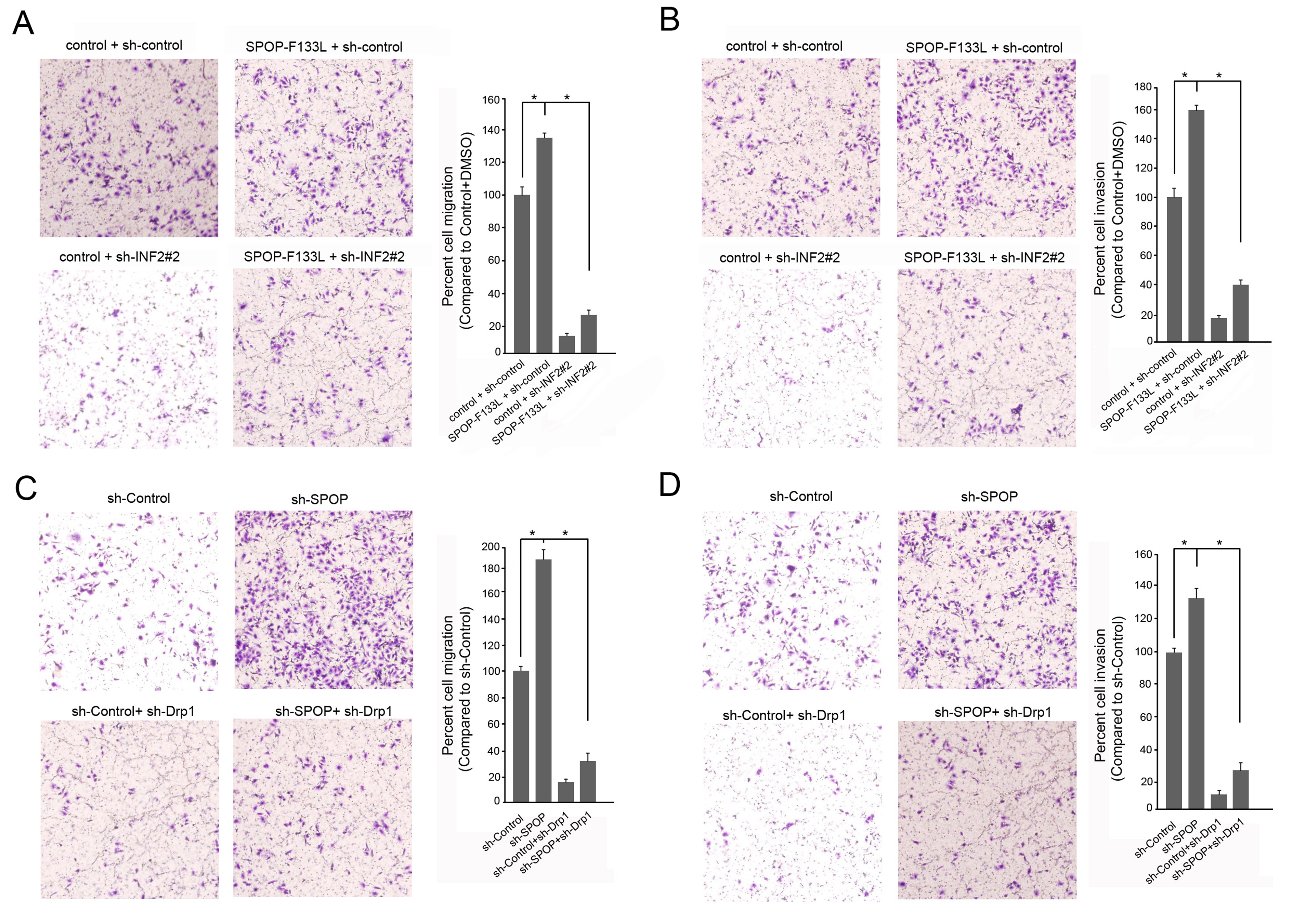

Supplement: S5 Fig — (A, B) DU145 cells were infected with lentivirus expressing HA-SPOP-F133L or control, then the stable cell lines were subsequent infected with lentivirus expressing sh-INF2 or sh-control. Cell migration assay (A) and invasion assay (B) were performed. *p < 0.01. (C, D) DU145 cells were infected with lentivirus expressing indicated shRNAs for cell migration and invasion assay. (TIF) [file pgen.1006748.s005.tif]

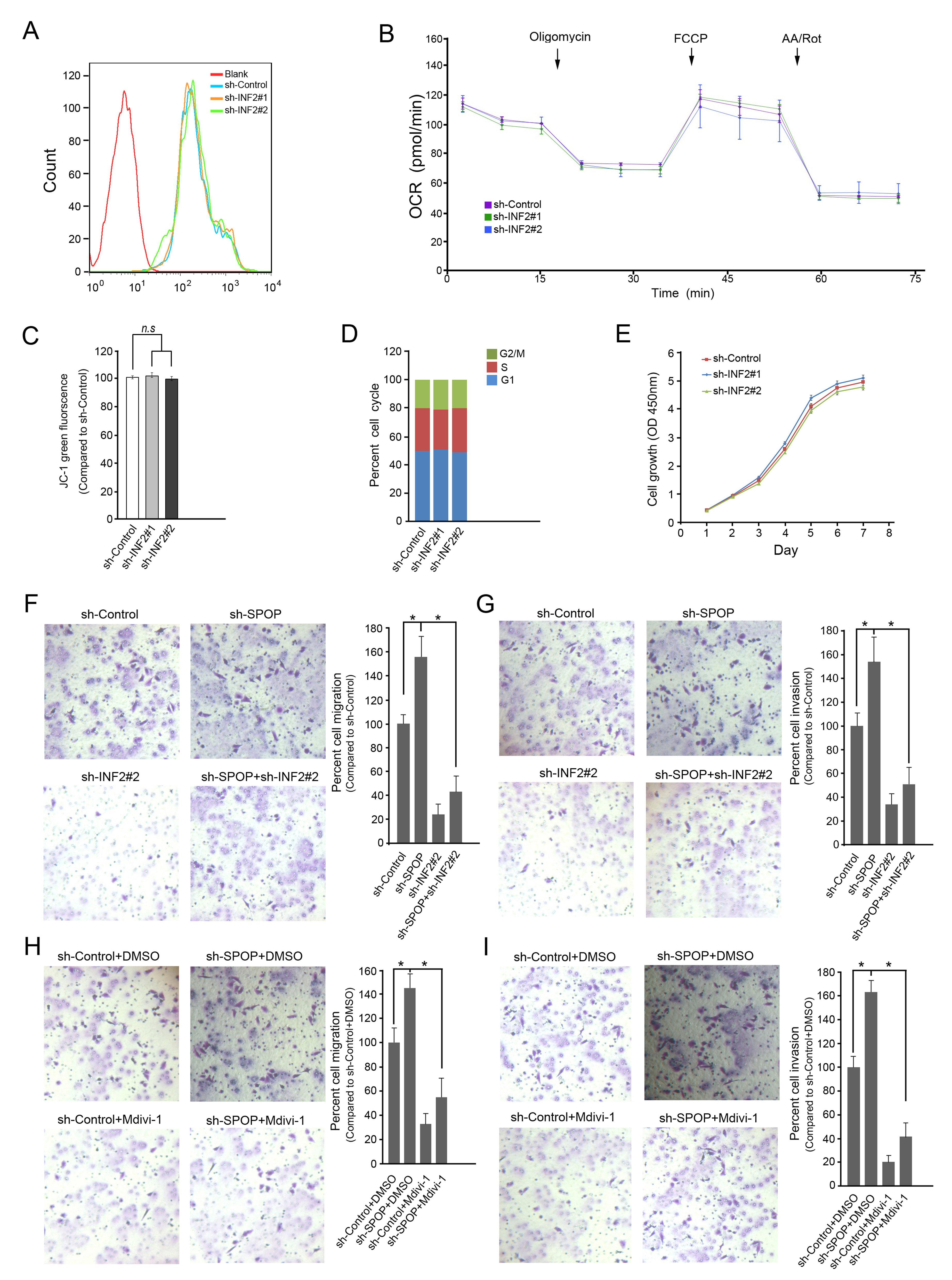

Supplement: S6 Fig — (A) MitoSOX Red was added to LNCaP cells expressing shRNA targeting INF2 or scramble control and fluorescence was measured by flow cytometry. (B) Oxygen consumption rate was measured using an XF24 extracellular flux analyzer in LNCaP cells expressing shRNA targeting INF2 or scramble control. Oligomycin, FCCP, Rotenone and Antimycin A were added at the indicated timepoints (arrows). Spare respiratory capacity is measured as the difference between basal oxygen consumption rate and the FCCP uncoupled oxygen consumption rate. (C) JC-1 fluorescent dye was added to LNCaP cells expressing shRNA targeting INF2 or scramble control. For quantification, the green fluorescence intensity (representing the degree of decreased ΔΨm) was measured by flow cytometry. Data represent three replicates. n.s, not statistically significant. (D) Cell cycle analysis of LNCaP cells infected with lentivirus expressing indicated shRNAs. (E) Cell growth analysis of LNCaP cells infected with lentivirus expressing indicated shRNAs. (F) LNCaP cells were infected with lentivirus expressing indicated shRNAs. Cell migration assay was shown on the left panel, and the quantitative analysis is shown on the right panel. All data shown are mean values ± SD (error bar) from three replicates. *p < 0.01 from triplicate. (G) LNCaP cells were infected with lentivirus expressing indicated shRNAs for cell invasion assay. (H) LNCaP cells were infected with lentivirus expressing indicated shRNAs and treated with Mdivi-1 (1 μM) for cell migration assay. (i) LNCaP cells were infected with lentivirus expressing indicated shRNAs and treated with Mdivi-1 (1 μM) for cell invasion assay. (TIF) [file pgen.1006748.s006.tif]

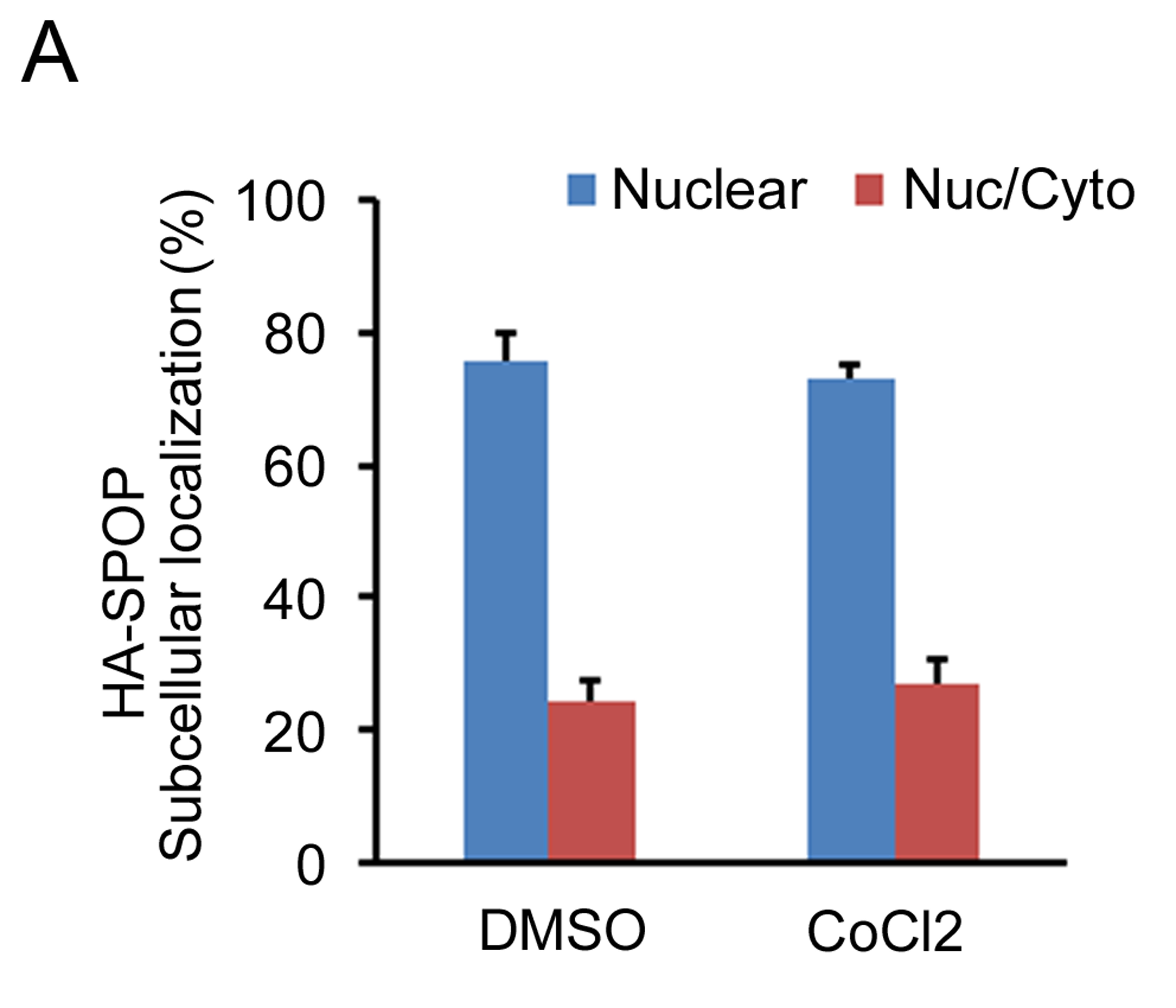

Supplement: S7 Fig — LNCaP cells infected with lentivirus expressing HA-SPOP, and treated with DMSO or CoCl2(100 μM) for 24hr. The relative Nuc/Cyto localization was quantified. n = 80 to 100 cells. Error bars, ± SD for triplicate. (TIF) [file pgen.1006748.s007.tif]
